# Competitive Endogenous Role of the LINC00511/miR-185-3p Axis and miR-301a-3p From Liquid Biopsy as Molecular Markers for Breast Cancer Diagnosis

**DOI:** 10.3389/fonc.2021.749753

**Published:** 2021-10-20

**Authors:** Marwa M. Mahmoud, Eman F. Sanad, Reham A.A. Elshimy, Nadia M. Hamdy

**Affiliations:** ^1^ Biochemistry Department, Faculty of Pharmacy, Ain Shams University, Cairo, Egypt; ^2^ Clinical and Chemical Pathology Department, National Cancer Institute, Cairo University, Cairo, Egypt

**Keywords:** LINC00511, breast cancer, competing-endogenous-RNA, molecular markers, liquid biopsy, miR-185-3p, miR-301a-3p, sponge theory

## Abstract

Breast cancer (BC) is the leading cause of female cancer-related mortalities. Evidence has illustrated the role of long non-coding RNAs (lncRNA) and microRNAs (miRNA) as promising pool of protein non-coding regulators, for tuning the aggressiveness of several malignancies. This research aims to unravel the expression pattern and the emphases of the diagnostic value of the long intergenic ncRNA00511 (LINC00511) and its downstream microRNA (miR-185-3p) and the pathogenic significance of the onco-miR-301a-3p in naïve BC patients. LINC00511 was chosen and validated, and its molecular binding was confirmed using bioinformatics. LINC00511 was measured in 25 controls and 70 patients using qPCR. The association between the investigated ncRNA’s expression and the BC patients’ clinicopathological features was assessed. Receiver operating characteristic (ROC) curve was blotted to weigh out their diagnostic efficacy over the classical tumor markers (TMs). Bioinformatics and Spearman correlation were used to predict the interaction between LINC00511, miR-185-3p, and miR-301a-3p altogether to patients’ features. LINC00511 and miR-301a-3p, in BC patients’ blood, were overexpressed, and their median levels increased significantly, while miR-185-3p was, in contrast, downregulated, being decreased fourfold. LINC00511 was elevated in BC early stages, when compared to late stages (*p* < 0.0003). LINC00511, miR-185-3p, and miR-301a-3p showed AUC superior to classical TMs, allowing us to conclude that the investigated ncRNAs, in BC patients’ liquid biopsy, are novel diagnostic molecular biomarker signatures. Lymph node metastasis (LNM) and advanced tumor grade were directly correlated with LINC00511 significantly. Additionally, both LINC00511 and miR-301a-3p were positively correlated with the aggressiveness of BC, as manifested in patients with larger tumors (>2 cm) at (*p* < 0.001). Therefore, these findings aid our understanding of BC pathogenesis, in the clinical setting, being related in part to the LINC00511/miR axis, which could be a future potential therapeutic target.

**Graphpical Abstract:**
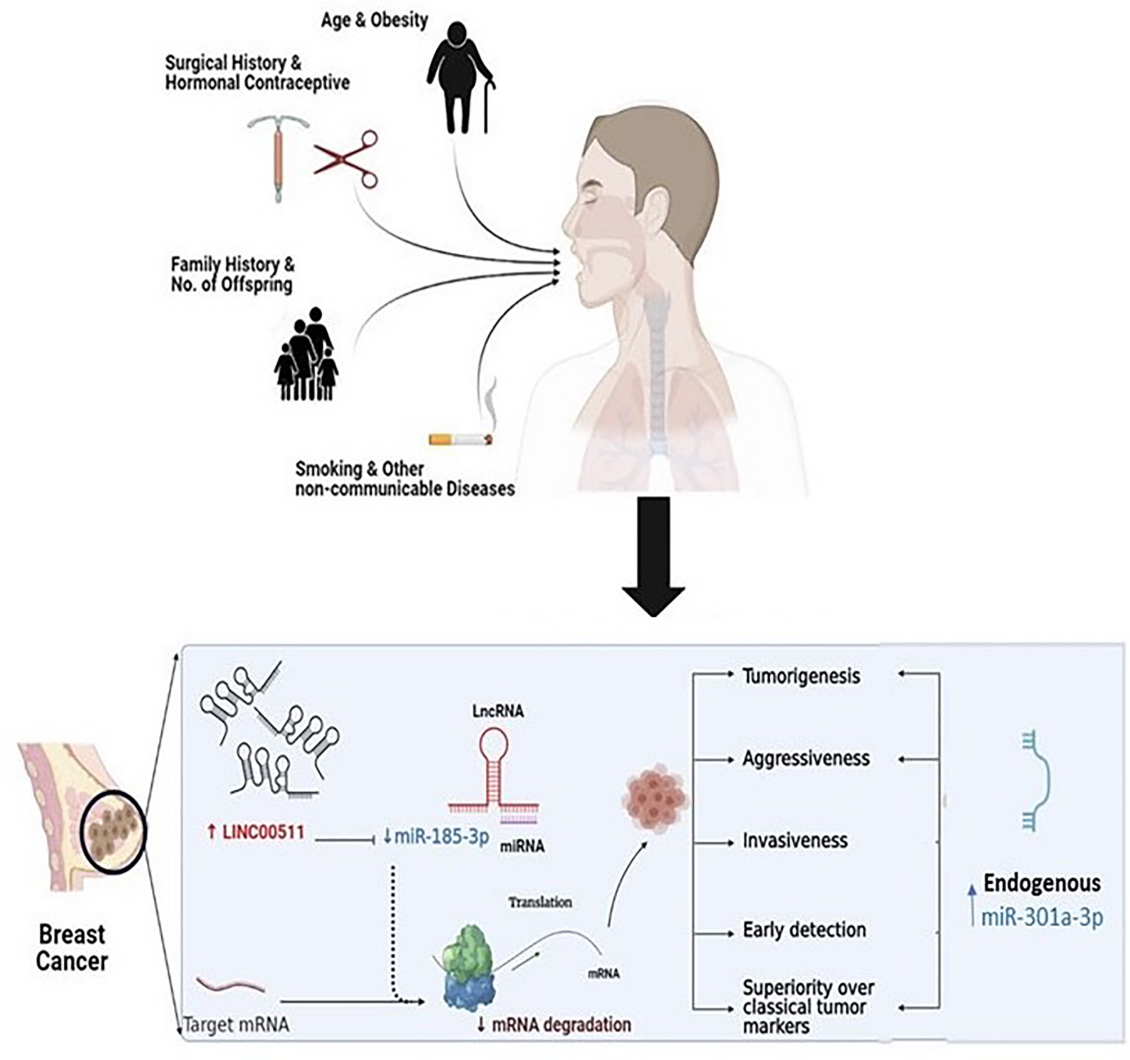


## Highlights

The fact that most previously identified diagnostic signatures of breast carcinoma (BC) are based on computed mammography, in addition to the classical protein-based tumor markers (TMs), whose expression levels are measurable in the advanced or late metastatic stages, with poor clinical outcome, is a crucial limitation. Therefore, the identification of molecular biomarker(s) obtained from BC patients’ liquid biopsy, with higher sensitivities than the classical TMs, is a global initiative we are working on for better earlier diagnostic results. A quantitative transcriptional analysis of protein non-coding RNAs (ncRNAs) was established to better diagnose early-stage (I/II) BC patients, in comparison to classical TMs. Hence, this would be a useful tool for BC clinical diagnosis.

## Introduction

Breast cancer (BC) is a major health concern impacting women globally. BC is the second most prevalent cancer among women (16% of all cancers) (1) and is the leading cause of cancer-related death in women around the world (2). BC accounts for 29% of the total patient cases at the National Cancer Institute (NCI), Cairo, Egypt (3). BC is a heterogeneous malignant tumor that varies widely between patients as well as within each single tumor; this is influenced by phenotypic (4) and genetic changes, with/without epigenetic modifications (5), hormonal receptors (6), and alteration, and is also affected by many biological influences as well as metabolic disturbances (7). Tumor heterogeneity is often related to late diagnosis and has a critical impact on patients’ clinical behavior, disease outcome, drug resistance, and poor patient survival (8). Delayed diagnosis with no clinical signs is mostly accompanied by weak prognosis and adverse outcome (9). Therefore, continuous search for an early diagnostic marker is ongoing, where non-invasive strategies for BC early diagnosis, as well as prognosis, are of prime importance, in order to sustain health, with better outcomes and decreased mortality (Sustainable development goals #3, health goal).

Sequencing technologies have successfully obtained a milestone in terms of non-coding RNAs (ncRNA) (10) as one of the most exciting biological discoveries in the past decade. Long-non-coding RNAs (LncRNAs) are a specific type of ncRNA made up of 200 nucleotides or more, which participates in various biological processes such as apoptosis (11), posttranscriptional processing (12), differentiation (13), chromatin remodeling (14), protein degradation and cell proliferation (15). Evidence connects lncRNA, expression changes, with several types of cancers (16–18), as BC progression (19) is either pro-oncogenic or suppressive. LncRNAs could behave as competitive-endogenous RNAs (ceRNAs) *via* engaging with microRNAs (miRNAs; miRs), thereby preventing miRNAs’ effects on their target messenger RNA (mRNA) (20); therefore, they are tumor suppressive.

Zhang et al. (21) implemented multi-RNA seq analyses of the lncRNA expression data, using the whole-transcriptome sequencing of 33 BC samples from The Cancer Genome Atlas Database to classify BC tissue-enriched lncRNAs, depicted as a heat map. This information was found in the database (DB) of NCBI GEO (Accession number GSE71651). We chose the long intergenic ncRNA 00511 (LINC00511, known also as LCAL5 or the onco-lncRNA-12), as the target of our research, after filtration and discussion. LINC00511, is a recently discovered oncogene, with a length of 2,265 bp, located on chromosome 17q24.3 and made up of five exons. It has been documented in different DBs, such as the LncBook Database, being dysregulated in glioma (22), ovarian cancer (23), cervical cancer (24), osteosarcoma (25), and lung cancer (26). LncRNA cellular localization is the first step in its mechanism of action (27), where cytoplasmic lncRNAs can affect mRNA stability or the cell signaling cascade (28). On the other hand, nuclear lncRNAs can modulate chromatin activity together with transcriptional regulation (29).

Lu et al. (30) found that LINC00511 was mechanically present in the BC stem cells’ cytoplasm, instead of the nucleus, assuming that LINC00511 mediates its downstream effects *via* post-transcriptional epigenetic changes, a point to be elucidated in the current research, where the precise regulatory role of LINC00511 in BC tumorigenesis is still clinically uncertain. Another question was raised, if mutual control exists between LINC00511 and some particular miRNA(s) in BC cases or not. Xu et al. (31) identified oncogenic lncRNAs involved in regulating the immune system activation and signaling pathways, where they identified the association between LINC00511 and progressing invasive BC subtype(s). This later subtype is of special interest as invasive BC subtype(s) are prevalent in Egypt.

MiRs are small regulatory ncRNAs of around 22 nucleotides length that regulate gene expression of target mRNAs, *via* suppressing their translation or decaying these translated targets (32). MiRNAs control about 30% of the human genome (20) and are similar to lncRNAs, contributing to BC metastasis (33).

Again, the regulatory axis lncRNA–miRNA–mRNA is now recognized, in which lncRNAs act as ceRNAs to inhibit miRNA transcription and their downstream biological functions, *via* suppression of miRNA targets (34). Based on bioinformatics analysis data using EMBOSS matcher and LncRNA SNP2 platform, we found that LINC00511 included the binding sequences matching the seed region of miR-185, a miR found on chromosome 22q11.21 (10), acting as an oncogene negative regulator or a tumor suppressor in osteosarcoma (35) and non-small cell lung cancer clinicopathological study (36). The precursor pre-miR-185 stem loop would give rise into two mature miRNAs called miR-185-5p and miR-185-3p strands, being present in either the forward or the reverse positions, respectively (37). According to the miRBase website, both miR-185-3p and miR-185-5p are stable strands.

Now, we can assume that LINC00511 and miR-185-3p are implicated in the clinical BC ceRNA network setting, a hypothesis previously proven in BC stem cells (30), but needs to be checked clinically.

Another miRNA prone to modifications in cancer is miR-301a, which is localized in the first intron of the spindle and kinetochore associated complex subunit 2 (SKA2) (38). miR-301a level was elevated in many solid cancers such as pancreatic (39), gastric (40), hepatocellular (41), and colorectal (38), and now to be investigated in BC patients. Again, miR-301a-3p level, and its correlation to LINC00511 expression in relation to tumor growth, proliferation, metastasis, and hormonal signaling, will be studied. According to the miRBase website, miR-301a-3p seems to be the most abundant and the most stable functional strand.

The current study aims to compare the expression level of LINC00511, miR-185-3p, and miR-301a-3p in both control and the BC patient groups’ blood samples as non-invasive molecular biomarkers for better BC diagnosis in female Egyptian patients’ liquid biopsy, in comparison to the classical diagnostic protein TMs. Second, it aims to explore LINC00511 influence on some BC hallmarks (tumor growth, proliferation, metastasis, and hormonal signaling). Third, it aims to investigate the effect of LINC00511 on miR-185-3p and miR-301a-3p, in an attempt to unravel its mechanistic function in BC clinically.

## Subjects

### Sample Size and Power Study

Based on the previous study by Hu et al. (42) with standard deviation (0.5) and large effect size (1.2), if the true differences between the BC group and the control group means are 4.47 and 3.20, respectively, the study group sizes are 25 patients and 25 control subjects. This is to be able to reject the null hypothesis that the population means of the studied groups are equal with a probability (power) of 0.9. Sample size estimation was performed by G power* sample size online calculator (http://www.gpower.hhu.de/en.html), depending on two-sided confidence level 95%.

### Study Participants

#### Ethical Approval and Consent to Participate

From October 2019 to November 2020, this case–control retrospective study was held. Ethical approvals were obtained from both the NCI, Cairo University, Ethical Committee and Ain Shams University, Faculty of Pharmacy’s review board Research Ethical Committee (REC ID 259, Date: September 9, 2019). The study was carried out according to the Declaration of Helsinki Guidelines; World Medical Association WMA Declaration of Helsinki: ethical principles for medical research involving human subjects, October 2013, revised July 2018, where all participating individuals (controls or patients) had signed a written detailed, ethically approved, informed consent.

The current retrospective cohort study included 70 female volunteer patients first diagnosed with primary BC, without any medical or surgical intervention, from the Clinical Oncology Department, NCI, Cairo University, Egypt. BC diagnosis is carried out with mammogram and MRI.

BC **patients’ inclusion criteria** were being an adult female, with breast invasive carcinoma of no specific type, confirmed pathologically. BC **patients’ exclusion criteria** were blood disease, any cancer other than BC, liver cirrhosis, and uterine and urinary bladder diseases, or metastatic BC patients who received chemo/radiotherapy, or had previous mastectomy.

### BC Patients’ Clinical and Pathological Features

For all BC participants, full family disease/cancer history was recorded, as well as patients’ previous surgical procedures, other than breast surgery, that do not affect the tumor burden, such as splenectomy, tonsillectomy, cesarean birth, and plastic surgery, offspring numbers, menopausal or not, and taking hormonal contraceptives or not.

Patients’ individual current cancer status and the tumor clinical assessment, done at the NCI, using the tumor-node-metastasis (TNM) categorization (43) and the Bloom–Richardson Scale for histological grading (44), were collected from patients’ data files, after a biopsy was taken at the time of BC examination.

Twenty-five healthy controls free from any medical, psychosocial, emotional conditions, or cancer (age interval was 28–82) were included in the study as well, matched with BC patients’ group in sex and race, menopausal status, and randomly chosen from the female Egyptian population.

ELISA results of the classical protein TM levels of carcinoembryonic antigen (CEA) and cancer antigen 15-3 (CA15-3), immunohistochemistry results for the proliferation index Ki-67 (either low or high), estrogen receptor (ER), progesterone receptor (PR) status, and human epidermal growth receptor 2 (HER2/neu) (positive or negative), patients’ weight and height for body mass index (BMI) calculation (https://www.nhlbi.nih.gov/health/educational/lose_wt/BMI/bmicalc.htm), age, and complete blood count (CBC) were all collected from patients’ files and tabulated for statistical analysis.

Molecular BC subtype (45) if luminal like (ER and/or PR positive), HER-2 overexpression (ER and PR negative, HER-2 positive), or triple-negative BC (TNBC) (ER negative, PR negative, and HER-2 negative) and histological BC subtypes, if invasive ductal carcinoma (IDC) or not, were all recorded for correlation analysis. BC patients were IDC (*n* = 59) cases, while the remainder (*n* = 11) had other forms of pathological invasive carcinoma. A total of 36 and 55 cancers cases were identified with an early stage (stages I–II) and low grade (grades I–II, according to Bloom–Richardson scale), respectively. Positive LNM, ER status, PR status, and HER-2/neu status were found in 34, 38, 50, and 23 BC patients, respectively.

## Methods

### *In Silico* Analysis

The webserver StarBase v2.0 (http://starbase.sysu.edu.cn) was utilized to identify promising lncRNA–miRNA interactions. The binding interaction was spotted using the bioinformatic software EMBOSS matcher.

### Blood Samples

Four milliliters of peripheral blood was withdrawn once from controls and BC patients, at the time patients were first diagnosed clinically with BC and before any medical (neo)adjuvant therapy or surgical intervention, under strict sterile conditions, following standard biosecurity and international safety procedures, into polymer gel vacutainers with a clot activator (Greiner Bio-One GmbH, Australia), left for 15 min at room temperature to clot, followed by a 10-min centrifugation at 10,000*g* at 4°C. Sera obtained were aliquoted into three clean Eppendorf tubes and stored at −80°C.

### Free ncRNA Extraction From Serum Samples and Purification Evaluation

Total RNA was extracted from serum samples (better yield than plasma) using the miRNeasy Mini kit (Catalog # 217004, Qiagen, USA). In brief, QIAzol lysis reagent (volume ratio 5:1) was applied to serum samples; one volume of chloroform was added followed by centrifugation for 15 min at 12,000*g* at 4°C using a cooling centrifuge (Hettich^®^ Universal 320/320R centrifuge). The upper aqueous phase was separated, and 1.5 volume of ice-cold absolute ethanol was added; 700 µl of the sample was pipetted into a RNeasy Mini column (Qiagen) and centrifuged for 15 s at room temperature. After washing the spin column with the washing buffer and eluting it with RNase-free water to a final volume of 30 µl, the purity and concentration of the RNA are determined using a Denovex^®^ DS-11 spectrophotometer (Wilmington, Delaware, USA). Samples were placed in aliquots at −80°C, until analysis. [N.B. ncRNA isolated as free circulating RNA in blood, not the encapsulated RNA within exosomes].

### Complementary DNA Synthesis and LINC00511 Expression Measurement Using Quantitative Real-Time Polymerase Chain Reaction

The RevertAid First Strand cDNA Synthesis Kit was used for cDNA synthesis (Thermo Scientific). As directed by the manufacturer, use a total volume of 20 µl of reverse transcription reaction components as follows: 1 µl random hexamer primer, 4 µl reaction buffer, 1 µl Ribolock RNase inhibitor, 2 µl dNTP mix, 1 µl RevertAid RT, 100 ng/ml purified lncRNA template, and RNase-free water to the final volume. The PCR carried out by Techne TC-3000G Thermal Cycler (San Diego, CA, USA) and the used protocol involved 5 min at 25°C, followed by 60 min at 42°C. The cDNA was stored at −20°C until the qPCR was performed.

In qPCR, SYBR green was used (5x HOT FIREPol^®^ Eva Green qPCR Mix Plus Kit). Five microliters of cDNA template was combined with 4 μl of Eva Green mixture, 2 μl of forward and 2 μl of reverse specific primers, and 7 μl of H_2_O. GAPDH Primer Assay was used as an endogenous control to normalize expression of investigated lncRNA. The thermal profile for qPCR was as follows: 12 min at 95°C for activation, followed by 40 cycles of denaturation at 95°C for 15 s, 60°C for 20 s, and 72°C for 20 s. Step-One Plus PCR detection system (Applied BioSystems, California, USA) was used to quantify LINC00511 expression using GAPDH as reference gene.

### cDNA Synthesis and miRNA Expression Measurement Using qPCR Reaction

For cDNA synthesis, the MiScript II reverse transcription kit (Cat # 218160, Qiagen) was used. A total volume of 20 µl of reverse transcription reagents was used as guided by the manufacturer protocol: 4 µl MiScript HiSpec buffer, 2 µl nucleic mixture, 2 µl MiScript RT mixture, RNase-free water (variable based on the amount of the supplied template miRNA), and pure miRNA template at a concentration of 100 ng/ml. To perform the transcription profiles, the PCR tubes were placed in a thermal cycler (Techne TC-3000G Thermal Cycler, CA, USA) for 60 min at 37°C. The cDNA was placed at −20°C until quantification.

qPCR was carried out using the MiScript primer assay (Cat number 218300, Qiagen) for miRNA-185-3p (hs-miR-185 MiScript Primer Assay, MS00008876) and miRNA-301a-3p (hs-miR-301a-3p MiScript Primer Assay, MS00009317) and the MiScript SYBR Green PCR kit (Cat number 218073, Qiagen). To normalize the expression levels of the investigated miRNAs, RNU6-2 or U6 (Hs RNU6-2_11 MiScript Primer Assay, MS00033740) was used as an endogenous control. The reaction mixture for the MiScript primer assays had a total volume of 20 µl. The thermal reaction condition was 40 cycles of 94°C for 15 s, 55°C for 30 s, and 70°C for 34 s, after an initial activation step for 15 min at 95°C. The qPCR was carried out by Step-One Plus PCR detection system (Applied BioSystems, Foster City, California, USA).

All primers are listed in **Table 1A**. RNA relative expression was computed and normalized as fold change using the CT cycle method (2^−ΔΔCt^) with GAPDH or U6 as the internal control for lncRNA and miRNA, respectively. ΔCt was determined by subtracting the Ct values of GAPDH and U6 from those of the LINC00511 and miRNAs under investigation, respectively. where ΔΔCt = ΔCt _cancer samples_ − ΔCt _control samples_ (46).

**Table 1A:** LINC00511, miR-185-3p, and miR-301a-3p primer sequences used for qPCR versus GAPDH and U6, respectively.

Target ID	Primer	Location	Amplicon size	Sequence (5` to 3`)
**LINC00511**	Forward	121–141	178 nt	5`-CTGTTTGGACGTGGTGAGGA-3`
	Reverse	319–338		5`-CCCTTCAGTTCATGACGCCT-3`
**GAPDH**	Forward	155–173	66 nt	5`-AGCCACATCGCTCAGACAC-3`
	Reverse	220–202		5`-GCCCAATACGACCAAATCC-3`
**miR-185-3p**	Forward	1–20	N.A.	5`-GGGGCTGGCTTTCCTCTGG-3`
	Reverse			N.A.
**miR-301a-3p**	Forward	1–22	N.A.	5`-CAGTGCAATAGTATTGTCAAA-3`
	Reverse			N.A.
**U6**	Forward	1–23	70 nt	5`-ATTGGAACGATACAGAGAAGATT-3`
	Reverse			5`-GGAACGCTTCACGAATTTG-3`

nt, nucleotides; N.A., not available to researchers, Intellectual property of Qiagen Co.

### Statistical Analysis

Data were tested for normality by SPSS 17.0 statistical package for social studies software (IBM, Armonk, NY). Normally distributed data are presented as mean ± S.E.M. Data are presented as median (range), if not normally distributed. Student’s *t*-test and ANOVA were used for comparison of two or more groups, if normally distributed, respectively. Mann–Whitney (*U*) or Kruskal–Wallis (*H*) was conducted to compare between any two or more independent groups, respectively. GraphPad prism was used to plot all the data graphically. Receiver operating characteristic (ROC) curve was performed to detect the best cutoff, sensitivities, specificities, negative predictive values (NPVs), and positive predictive values (PPVs), with an AUC calculated. A ROC curve was performed using MedCalc Statistical Software version 19.2.6 (MedCalc Software by Ostend, Belgium) (https://www.medcalc.org) between BC patients and healthy control individuals to detect the sensitivities and the specificities for the protein-based TMs, lncRNA, and miRNAs investigated and their clinical efficacy. Negative likelihood ratios (LRs) in medical testing are used to interpret the diagnostic tests. Basically, the LR tells how likely a patient has a disease or condition. The higher the ratio, the more likely they have the disease or condition, confirming the obtained sensitivities and specificities from the ROC curve. Sensitivity and specificity are an alternative way to define the likelihood ratio, where negative LR = (100 – sensitivity)/specificity.

Multiple regression analyses were run to investigate the influence of age, BMI, menopausal status, BC family history, pt. surgical history, hemoglobin content, platelet count, and number of offspring (independent variables) on the ncRNA expression levels as dependent variables. Correlation between different variables was assessed by Spearman’s correlation coefficient *r*. Also, the expression level of LINC00511 and the “investigated miRs” were set to Spearman correlation, while point-biserial correlation was used to measure the association that exists between two variables, one continuous and one dichotomous. *p*-values were two-tailed and considered significant if *p* ≤ 0.05.

## Results

### Bioinformatics Analysis

MiRnet platform was used to identify miRs implicated in the BC network. GSM4700099 was the accession number for the RNA-seq data obtained from the GEO repository (BC patients). The overlap of gene sets of interest with annotated gene sets deposited in the Molecular Signatures Database (MSigDB) was computed for gene set enrichment analysis v6.0. (Broad Institute, Cambridge, Massachusetts, USA). Using the miRbase.org database (http://www.mirbase.org) and the RefSeq database (https://www.ncbi.nlm.nih.gov/nuccore/NR_033876.1), miR-185-3p, miR-301a-3p, and LINC00511 accession numbers and mature sequences were retrieved (**Table 1B**).

**Table 1B:** LINC00511, miR-185-3p, and miR-301a-3p accession number, chromosome, cytogenetic band, size bases, strand, mature sequence, and sequence size obtained from databases (DB).

RNA Gene DB ID	Accession number/Chromosome; Cytogenetic band	Pre-ncRNA Size bases/Strand	Mature Sequence (5` to 3`)/Size
**LINC00511**	URS00001710A5_9606	350,382	72,290,091-AAGGGGCGAUGCGCCCCGGAGGGGGA//CAGAUUCUGAUGCUCGGUGGACAGU-72,640,472
	Chr17; 17q24.3	Reverse (−)	2,265
**miR-185-3p**	MIMAT0004611	82	50 - AGGGGCUGGCUUUCCUCUGGUC - 71
	Chr22; 22q11.21	Forward (+)	21
**miR-301a-3p**	MIMAT0000688	86	51-CAGUGCAAUAGUAUUGUCAAAGC-73
	Chr17; 17q22	Reverse (−)	22

miRGene DB was used for both miR-185-3p and miR-301a-3p, and RefSeq DB was used for LINC00511.

### Participants’ Clinical and Demographic Characteristics

The present research comprised a total of 95 participants matching the inclusion criterion. They were 70 patients with BC and 25 control group of healthy females; their mean age in years ± S.E.M. was 50.01 ± 1.46 and 49.8 ± 2.38, respectively, with no significant differences, while a significant difference was reported between the two groups regarding BMI, hemoglobin (Hb), and platelet count. Premenopausal status was reported in 40 BC cases (57.1%) and 17 (68.0%) healthy female participants, respectively. In BC patients, CEA and CA15-3 median levels were 2.39 ng/ml and 23.8 U/ml, respectively, compared to their median levels in the control group (1.4 ng/ml and 6.8 U/ml, at *p* = 0.155 and 0.0001, respectively) (**Table 2**).

**Table 2:** Study participants’ clinical and demographic features and targeted ncRNA expression levels.

Groups (*n*)/Characteristics (Unit)	Control (25)	BC patients (70)	Statistics
**Age (years)**			
≤50 (*n*, %)	(10, 40%)	(39, 55.7%)	*χ* ^2^ = 1.821, *p*1 = 0.244
>50 (*n*, %)	(15, 60%)	(31, 44.3%)	
**BMI (kg/m^2^)**			
<25 (*n*, %)	(20, 80%)	(3, 4.3%)	N.A
≥25 (*n*, %)	(5, 20%)	(32, 45.7%)	
N.A (*n*, %)	(0, 0%)	(35, 50%)	
**No. of offspring**			
≤2 (*n*, %)	(17, 68%)	(38, 54.3%)	*χ* ^2^ = 1.42, *p*1 = 0.233
>2 (*n*, %)	(8, 32%)	(32, 45.7%)	
**TLC (×10^3^ cell/µl)**			
≤5 (*n*, %)	(20, 80%)	(25, 35.7%)	
>5 (*n*, %)	(5, 20%)	(45, 64.3%)	
**Hemoglobin (gm/dl)**			
≤10 (*n*, %)	(1, 4%)	(54, 77.1%)	
>10 (*n*, %)	(24, 96%)	(16, 22.9%)	
**Platelet count (×10^3^cell/µl)**			
≤150 (*n*, %)	(2, 8%)	(12, 17.2%)	
>150 (*n*, %)	(23, 92%)	(58, 82.8%)	
**Menopause**			
Pre (*n*, %)	(17, 68%)	(40, 57.1%)	*χ* ^2^ = 0.904, *p*1 = 0.341
Post (*n*, %)	(8, 32%)	(30, 42.9%)	
**Non-Communicable Diseases**			
C.V.D. (*n*, %)	(0, 0%)	(32, 45.7%)	N.A
D.M. (*n*, %)	(0, 0%)	(9, 12.9%)	
None (*n*, %)	(25, 100%)	(29, 41.4%)	
**BC Family History**			
No (*n*, %)	(25, 100%)	(54, 77.1%)	
Yes (*n*, %)	(0, 0%)	(16, 22.9%)	
**Pt. Surgical History**			
No (*n*, %)	(25, 100%)	(40, 57.1%)	N.A
Yes (*n*, %)	(0, 0%)	(30, 42.9%)	
**Hormonal Contraceptive Intake**			
No (*n*, %)	(25, 100%)	(53, 75.7%)	N.A
Yes (*n*, %)	(0, 0%)	(17, 24.3%)	
**CEA (ng/ml)**	1.4 (0.99–1.87)	2.39 (0.3–6.6)	*NS*
**CA15-3 (U/ml)**	6.8 (4.3–17.2)	23.8 (6.3–90.6)	*p*2 < 0.0001*
**LINC00511 (Fold change)**	0.008 (0.0002–0.97)	2.5 (0.018–21.77)	*p*2 = 0.006*
**miR-185-3p (Fold change)**	2.14 (0.53–3.04)	0.55 (0.01–2.76)	*p*2 = 0.0001*
**miR-301a-3p (Fold change)**	0.24 (0.01–1.66)	3.4 (0.14–20.6)	*p*2 = 0.0004*

Data shown as mean ± S.E.M. or median (range) or (n, %), Statistics were computed using GraphPad prism software using Chi square test χ2 (dichotomous parameters), Student’s t-test (parametric data), or Mann–Whitney test (non-parametric data), p1 indicates comparison between number of populations in control and BC groups using Chi-square test, while p2 indicates comparison between mean or median of population in control and BC groups, *Statistical significance p-value < 0.05. [BMI, body mass index; C.V.D., cardiovascular diseases; D.M., diabetes mellitus; TLC, total leucocyte count; N.A., not applicable/not available; NS, non-significant].

### Expression Levels of the Investigated ncRNAs

**Figure 1:**
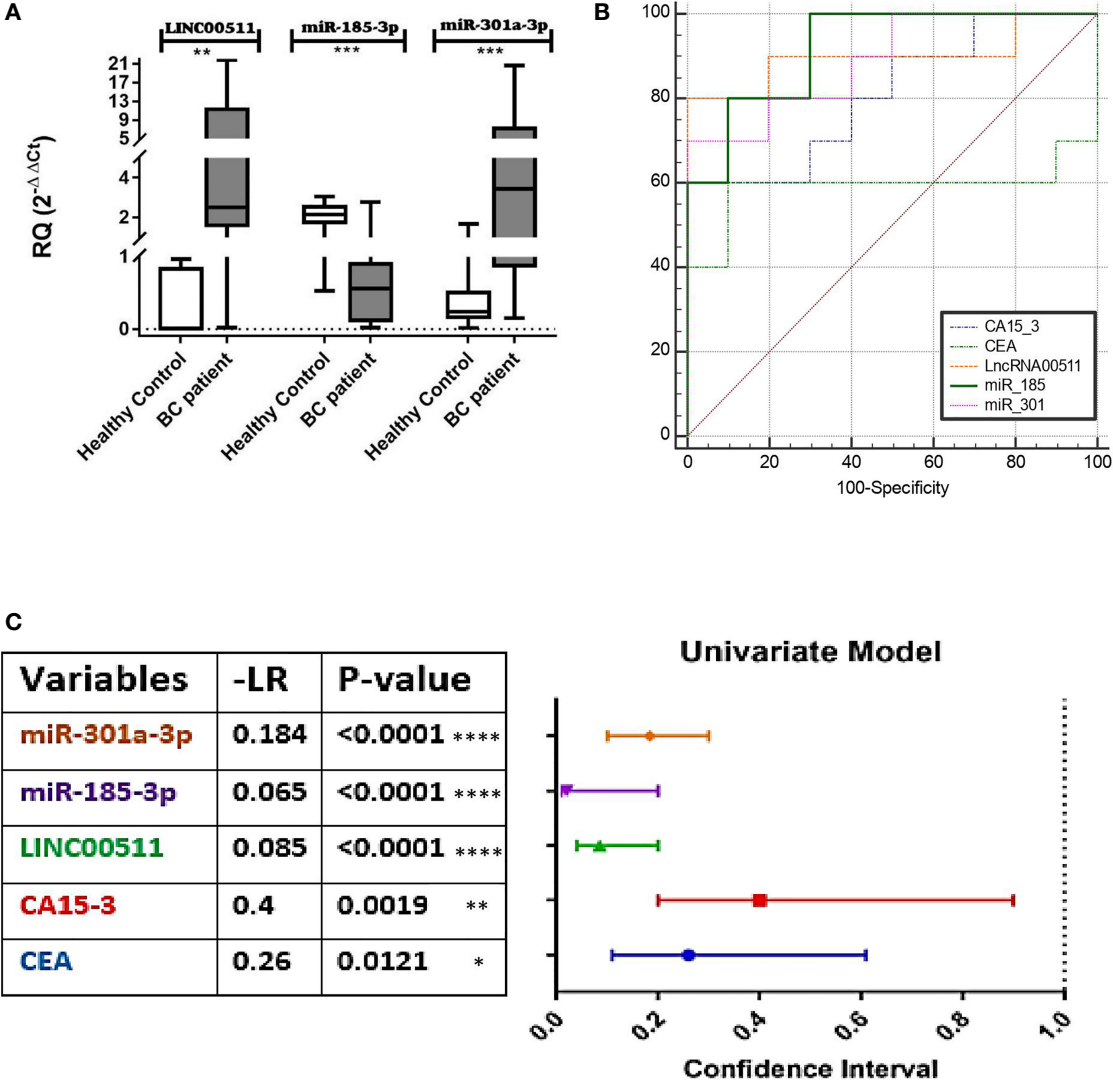
Diagnostic value of LINC00511, miR-185-3p, and miR-301a-3p. **(A)** Expression levels of targets in BC patients. LINC00511 and miR-301a-3p were significantly overexpressed, and on the contrary, miR-185-3p was reciprocally downregulated in BC patients, **(B)** receiver operating characteristic (ROC) curves for the investigated biomarkers, **(C)** Negative Likelihood Ratios (−LR) of the estimated markers; estimated % decrease in probability is large (45%) when −LR = 0.1, moderate (30%) when −LR = 0.1–0.2, and small (15%) when −LR = 0.2–0.5. [All values were obtained using MedCalc software (https://www.medcalc.org/calc). Data were plotted graphically using GraphPad prism software]. ****p < 0.0001. ***p < 0.001. **p < 0.01. *p < 0.05. RQ, relative expression; BC, breast cancer.

qPCR was used to detect LINC00511, miR-185-3p, and miR-301a-3p expression levels in BC patients and control sera. LINC00511 and miR-301a-3p expression were significantly upregulated (*p* ≤ 0.006 and 0.0004, respectively), whereas miR-185-3p expression was significantly downregulated in BC blood samples (*n* = 70) in comparison to the control group (*n* = 25) (*p* ≤ 0.0001) as shown in **Figure 1A**.

In BC patients, LINC00511 and miR-301a-3p median levels were 2.5-fold change and 3.4-fold change, respectively. These median levels of LINC00511 and miR-301a-3p in BC patients were higher by 312-fold and 14.5-fold, respectively, in comparison to the control group. On the other hand, miR-185-3p median level was 0.55-fold change, which is fourfold decreased (*p* = 0.0001) when compared to the healthy control group (**Table 2**).

### Diagnostic Efficacy for the Investigated Molecular Biomarkers in Comparison to Protein-Based Conventional BC TMs

ROC curve analysis for the individual investigated markers was done to examine the diagnostic capacity/utility of the investigated molecular biomarkers in comparison with CEA and CA15-3 for better BC diagnosis, *via* comparing using fixed cutoff values. The AUC of the ROC curves ranged between 0.759 and 0.98. The cutoff values that discriminate between BC patients and the control individuals detected an absolute specificity of 100% with a sensitivity of 91.43% for LINC00511, a 78.57% specificity and an 85.51% sensitivity for miR-301a-3p, and a 66.67% specificity and a 95.65% sensitivity for miR-185-3p. For the classical BC protein TMs, specificities and sensitivities were 90% and 100%, and 76.47% and 60% for CEA and CA15-3, respectively. A significant increase in the diagnostic specificity (96.67%) was found, if we combine CEA and miR-185-3p, as tumor markers, with AUC 0.92 (95% CI: 0.709 to 0.993, *p* = 0.0183) as presented in **Figure 1B** and **Table 3**.

**Table 3:** Overall sensitivities, specificities, and predictive values for the tested markers (molecular; ncRNAs and protein TMs) in BC patients (*n* = 70) single or combined.

	Single					Combined
Markers (Units)	CEA (ng/ml)	CA15-3 (U/ml)	LINC00511 (Fold Change)	miR-185-3p (Fold Change)	miR-301a-3p (Fold Change)	CEA + miR-185-3p
**Cutoff value**	>1.66	>17.2	>0.97	≤1.74	>0.59	-
**AUC**	0.759	0.810	0.980	0.838	0.899	0.92
**S.E.M.**	0.103	0.0999	0.0114	0.0641	0.0399	0.0589
***p*-value**	0.01*	0.00*	0.000*	0.000*	0.000*	0.0183*
**95% CI**	0.557–0.901	0.575–0.948	0.925–0.998	0.740–0.911	0.813–0.954	0.709–0.993
**Sensitivity %**	76.47	60.00	91.43	95.65	85.51	73.19
**Specificity %**	90	100	100.00	66.67	78.57	96.67
**PPV**	60	100	100	36.01	43.9	0.145
**NPV**	95.12	92.7	98.34	98.73	96.5	4.72
**Accuracy**	0.886	0.795	0.943	0.875	0.784	92.82

Data were obtained from the ROC curve analysis using MedCalc software. [AUC, area under the curve; PPV, positive predictive value; NPV, negative predictive value, *Statistically significant p-value < 0.05].

**Figure 1C** presents the negative likelihood ratio following the rule of thumb (McGee, 2002; Sloane, 2008) where 0 to 1: decreased evidence for disease. Values closer to zero have a higher decrease in probability of disease (meaning increased marker sensitivity). This confirms that the best calculated sensitivities and specificities was for miR-185-3p, followed by LINC00511, miR301-3p, and, finally, CEA, from the best cutoff obtained for each, after the significant AUC from the ROC curve (**Figure 1B** **).**

Different significant levels were detected among the studied groups, when the cutoff values of the measured markers were considered. As shown in **Table 4**, the value was considered positive or negative for the marker, whether it was above or below the cutoff value. In BC patients with positive rates regarding the cutoff value, CEA, CA15-3, LINC00511, and miR-301a-3p median levels were 2.9 ng/ml, 32.4 U/ml, 2.02-fold change, and 3.9-fold, respectively, while at the cutoff value ≤1.7-fold, miR-185-3p median level in the BC patients with positive rates was 0.47-fold change.

**Table 4:** Cutoff values and median levels with positive rates of classical TMs and circulating ncRNAs under investigation in control (*n* = 25) and BC patients’ group (*n* = 70).

Groups	TMs				ncRNAs (Fold Change)					
	CEA (ng/ml)		CA15-3 (U/ml)		LINC00511		miR-185-3p		miR-301a-3p	
**Cutoff**	>1.66	Median	>17.2	Median	>0.97	Median	≤1.7	Median	>0.59	Median
**Control**	2 (8%)	1.7 (1.6–1.8)	0 (0%)	–	0 (0%)	–	4 (16%)	0.67 (0.5–0.79)	3 (12%)	0.67 (0.591–1.6)
**BC**	53 (75.7%)	2.9 (1.6–6.6)	49 (70%)	32.4 (20.3–90.6)	64 (91.4%)	2.02 (1.07–8.6)	64 (91.4%)	0.47 (0.01–1.6)	60 (85.7%)	3.9 (0.6–20.6)

All data are presented as median (range) and the positivity rates are presented as (n, %).

Based on the sensitivity, specificity, PPV, and NPV obtained from the ROC curve, we discovered that LINC00511 and miRNAs were superior for BC diagnosis and, moreover, showed greater sensitivities than the classical protein TMs for BC early-stage detection, as clearly seen in **Table 3**.

### Relations Between the Investigated Molecular Biomarkers and Patient Clinicopathological Factors

**Figure 2:**
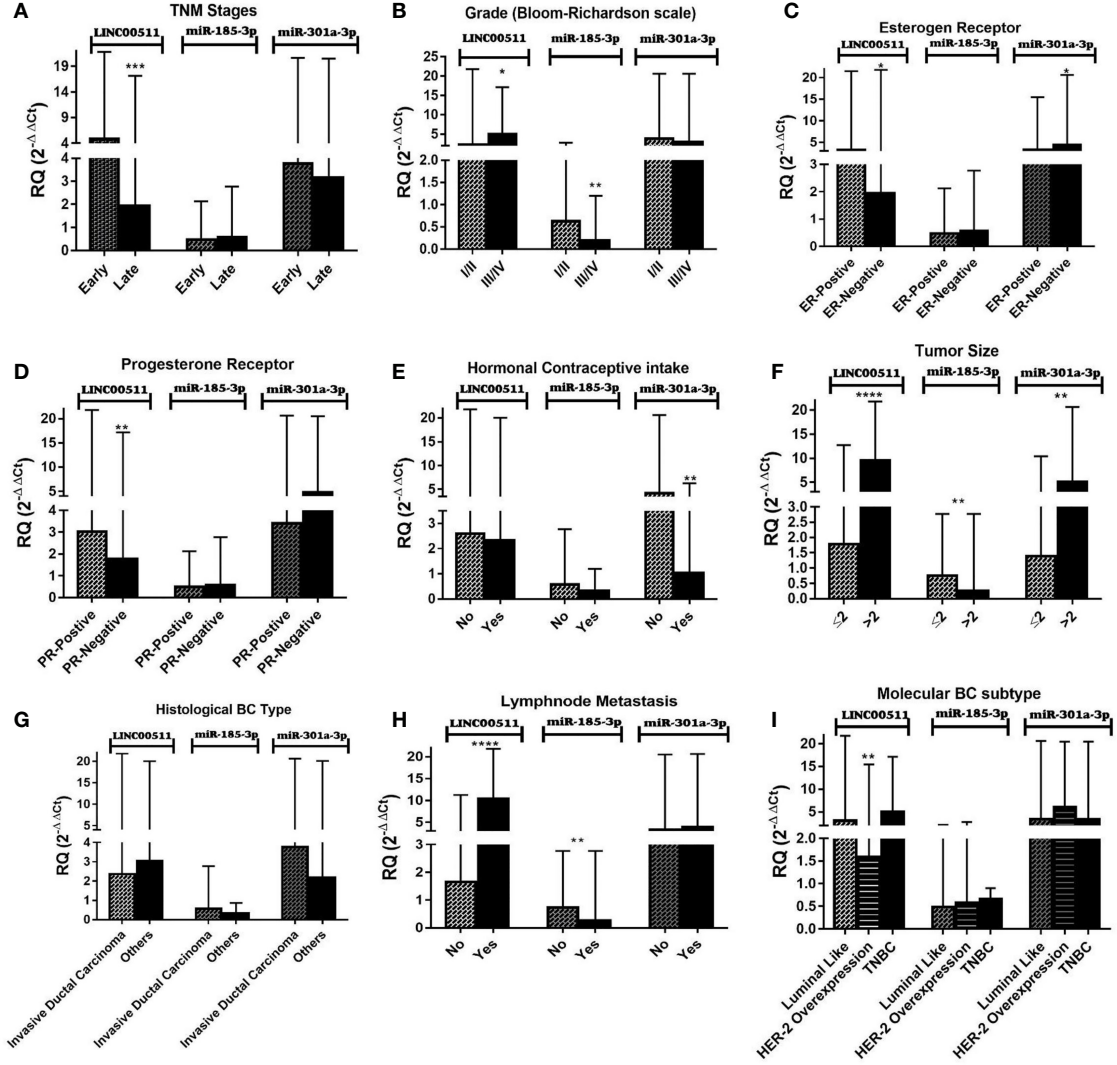
Impact of clinicopathological features on LINC00511, miR-185-3p, and miR-301a-3p expression in BC patients. **(A)** Early and late stage (TNM, tumor node metastasis), **(B)** tumor grades (Bloom–Richardson scale), **(C)** estrogen receptors, **(D)** progesterone receptors, **(E)** hormonal contraceptive intake, **(F)** tumor size, **(G)** histological BC type, and **(H)** lymph node metastasis. **(I)** Molecular BC subtypes. [The Mann–Whitney test was performed. Data are presented as Median (Range). ****p < 0.0001, ***p < 0.001, **p < 0.01, *p < 0.05, compared with other breast cancer (BC) subgroups using GraphPad prism software].

It is worth noting that there is no clear significant relation between protein-based TMs (CEA and CA15-3) and the patients’ clinicopathological features in our study. **Figure 2** and **Table 5** present the association between the investigated molecular biomarkers with various clinicopathological factors. There is a significant difference between LINC00511 expression with the TNM clinical stages and histological grading. The expression of LINC00511 with hormonal receptor status was significant for ER status, PR status, tumor size, LNM, and BC molecular subtypes. Also, miR-301a-3p showed significant overexpression for negative ER status, as compared to positive ER status. In addition, its median level decreased significantly in patients receiving hormonal contraceptives, while miR-185-3p reported a significant difference with histological grading as well as both tumor size and LNM, where its expression was decreased in the advanced grade cases, tumors with larger sizes, and positive LNM.

**Table 5:** Expression levels of LINC00511, miR-185-3p, and miR-301a-3p in BC patient (*n* = 70) stratified group/subgroups.

Characteristics		BC patients		LINC00511	miR-185-3p	miR-301a-3p
		*n*	%	Median (range)	Median (range)	Median (range)
**Age (years)**				
≤50		39	55.7	2.36 (0.01–21.48)	0.48 (0.01–2.76)	2.98 (0.14–20.4)
>50		31	44.3	3.16 (0.65–21.78)	0.62 (0.03–1.74)	3.75 (0.46–20.6)
*p-*value				NS	NS	NS
**No. of offspring**						
≤2		38	54.3	2.75 (0.65–20.11)	0.4 (0.02–1.74)	3.75 (0.14–20.4)
>2		32	45.7	2.19 (0.01–21.78)	0.63 (0.01–2.76)	3.37 (0.14–20.6)
*p-*value				NS	NS	NS
**Non-Communicable Diseases**						
C.V.D		32	45.7	2.18 (0.01–21.78)	0.618 (0.0166–2.769)	3.152 (0.444–20.62)
D.M		9	12.9	2.33 (1.559–11.26)	0.303 (0.0718–1.74)	3.361 (0.149–20.46)
None		29	41.4	2.69 (0.41–21.48)	0.323 (0.0166–2.769)	3.756 (0.149–20.48)
*p-*value				NS	NS	NS
**BC Family History**						
No		54	77.1	2.31 (0.65–21.78)	0.5682 (0.0166–2.769)	3.338 (0.149–20.62)
Yes		16	22.9	4.17 (0.018–21.48)	0.3009 (0.0166–2.769)	5.434 (0.323–20.62)
*p-*value				NS	NS	NS
**Pt. Surgical History**						
No		40	57.1	2.51 (0.018–21.78)	0.5566 (0.0219–2.769)	3.432 (0.149–20.62)
Yes		30	42.9	2.65 (0.41–21.48)	0.5689 (0.0166–2.128)	3.559 (0.323–19.69)
*p-*value				NS	NS	NS
**Hormonal Contraceptive intake**						
No		53	75.7	2.59 (0.018–21.78)	0.5701 (0.0349–2.769)	4.225 (0.149–20.62)
Yes		17	24.3	2.33 (1.22–20.04)	0.3207 (0.0166–1.197)	1.027 (0.149–6.273)
*p*-value				NS	NS	U=232; *P*= 0.0023*
**TNM Stage**						
I-II (early stage)		36	51.4	4.79 (0.73–21.78)	0.474 (0.034–2.12)	3.75 (0.154–20.62)
III (late stage^$^)		34	48.6	1.92 (0.018–17.16)	0.576 (0.0166–2.76)	3.15 (0.14–20.48)
*p*-value				*U* = 313; *p* = 0.0003*	NS	NS
**Tumor Size (cm)**						
≤ 2		35	50	1.792 (0.018–12.73)	0.747 (0.0166–2.76)	1.39 (0.149–10.4)
> 2		35	50	9.52 (0.41–21.78)	0.251 (0.016–2.76)	5.09 (0.46–20.62)
*p*-value				*U* = 138; *P*< 0.0001*	*U* = 381; *p* = 0.0062*	*U* = 339; *p* = 0.0011*
**Grade (Bloom–Richardson scale)**						
I/II		55	78.6	2.29 (0.018–21.78)	0.623 (0.02–2.76)	3.75 (0.15–20.62)
III/IV		15	21.4	4.93 (1.97–17.16)	0.188 (0.01–1.19)	2.9 (0.149–20.48)
*p*-value				*U* = 263; *p* = 0.031*	*U* = 222; *p* = 0.0056*	NS
**LNM**						
No		36	51.4	1.65 (0.417–11.26)	0.727 (0.0166–2.76)	3.33 (0.32–20.48)
Yes		34	48.6	10.41 (0.018–21.78)	0.25 (0.0166–2.769)	3.99 (0.14–20.62)
*p*-value				*U* = 82; *p* < 0.0001*	*U* = 373; *p* = 0.0045*	NS
**Histological BC type**						
IDC		59	84.2	2.363 (0.018–21.78)	0.57 (0.01–2.76)	3.75 (0.14–20.62)
Other types		11	15.8	3.033 (1.23–20.04)	0.32 (0.03–0.87)	2.18 (0.32–20.11)
*p*-value				NS	NS	NS
**BC Molecular subtype**				
Luminal like		51	72.8	3.03 (0.66–21.78)	0.4828 (0.0166–2.128)	3.361 (0.149–20.62)
HER-2 overexpression		16	22.8	1.59 (0.018–15.43)	0.5761 (0.0166–2.769)	6.023 (0.444–20.46)
TNBC		3	4.4	4.93 (1.65–17.16)	0.6549 (0.054–0.9009)	3.315 (0.641–20.48)
*p*-value				*H*= 9.2; *p* = 0.0098*	NS	NS
**ER status**						
Positive		38	54.3	3.27 (0.66–21.48)	0.47 (0.03–2.12)	3.36 (0.14–10.4)
Negative		32	45.7	1.94 (0.018–21.78)	0.57 (0.01–2.76)	4.42 (0.32–20.62)
*p*-value				*U* = 428; *p* = 0.0337*	NS	*U* = 423; *p* = 0.0417*
**PR status**						
Positive		50	71.4	3.02 (0.66–21.78)	0.486 (0.016–2.12)	3.39 (0.14–20.62)
Negative		20	28.6	1.63 (0.018–17.16)	0.576 (0.01–2.76)	4.76 (0.149–20.48)
*p*-value				*U* = 317; *p* = 0.0039*	NS	NS
**HER‐2/neu status**						
Positive		23	32.9	2.01 (0.018–21.78)	0.566 (0.016–2.12)	3.36 (0.149–20.62)
Negative		47	67.1	2.69 (0.655–20.04)	0.323 (0.0219–2.76)	3.75 (0.323–20.62)
*p*-value				NS	NS	NS
**Ki-67 status**						
High		10	14.2	2.865 (1.19–21.48)	0.23 (0.04–1.08)	2.98 (0.46–7.3)
Low		3	4.3	17.16 (1.65–20.04)	0.65 (0.46–0.9)	1.02 (0.64–3.31)
N.A		57	81.5	–	–	–
*p*-value				NS	NS	NS
**CEA (ng/ml)**						
Cutoff ≤1.66		17	24.3	4.38 (0.41–21.78)	0.30 (0.016–2.76)	4.75 (0.14–20.62)
Cutoff >1.66		53	75.7	2.36 (0.018–20.11)	0.62 (0.03–0.9)	3.31 (0.1–20.6)
*p*-value				NS	NS	NS
**CA15-3 (U/ml)**						
Cutoff ≤17.2		21	30	2.59 (1.47–16.87)	0.57 (0.016–2.12)	4.75 (0.32–20.62)
Cutoff >17.2		49	70	2.42 (0.01–21.78)	0.48 (0.01–2.71)	3.43 (0.14–20.6)
*p*-value				NS	NS	NS

All data were expressed as median (range) and comparison was assessed using Mann–Whitney test (U) for comparison of two non-parametric groups and Kruskal–Wallis one-way ANOVA (H) for more than two non-parametric groups on GraphPad prism software. *Significant statistical difference less than 0.05, P statistical difference between BC subgroups, ^$^late localized advanced stage [CVD, coronary vascular diseases; DM, diabetes mellitus; ER, estrogen receptor; HER-2, human epidermal growth factor receptor-2; IDC, invasive ductal carcinoma; LNM, lymph node metastasis; Ki-67, proliferative index; PR, progesterone receptor; TNBC; triple-negative breast cancer; TNM, tumor node metastasis; NS, Non Significant].

The correlations within LINC00511, miR-185-3p, and miR-301a-3p and clinicopathological characteristics were evaluated as well. miR-301a-3p expression level was inversely correlated with pt. surgical history, while no significant correlations were detected between LINC00511, miR-185-3p, and miR-301a-3p expression and other clinicopathological features including age, menopausal status, tumor size, ER, PR, and HER-2/neu status in the BC patients. Detailed correlation information is provided in **Supplementary Table S1**. In addition, a multiple regression was run to investigate the influence of age, BMI, menopausal status, BC family history, pt. surgical history, hemoglobin content, platelets count, and number of offspring, as independent variables, on the expression levels of LINC00511, miR-185-3p, or miR-301a-3p (dependent variables). These variables were not significant predictors for either ncRNA (**Supplementary Table S2**).

### miR-185-3p as a Target for LINC00511 in BC cells

**Figure 3:**
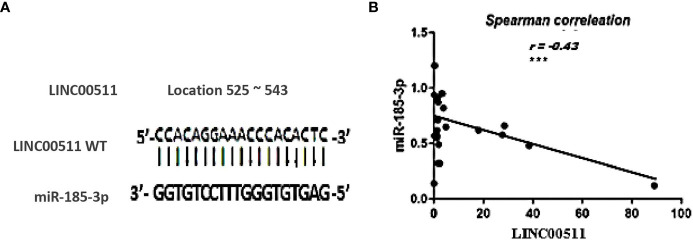
The relationship between LINC00511 and miR-185-3p in BC patients (*n* = 70). **(A)** the predicted potential binding regions between LINC00511 WT (top) and miR-185-3p (bottom). **(B)** Spearman correlation analysis of LINC00511 and miR-185-3p. ***p < 0.001 [WT, wild type].

In an attempt to investigate the relationship of LINC00511 with other ncRNAs using bioinformatic analysis, miR-185-3p was found to hit the LINC00511 525 to 543 sequence with high score = 80% (**Figure 3A**).

The Cancer Genome Atlas dataset in starBase, a bioinformatic tool, predicted a possible inverse correlation between LINC00511 and miR-185-3p expression level. Moreover, when using the EMBOSS matcher software, LINC00511 contained binding sequences complementary (13/19 bases: 74%) to miR-185-3p seed regions, as shown in **Figure 3A**. To validate the online prediction even more, Spearman correlation analysis was done and showed a significant inverse correlation between LINC00511 and miR-185-3p expression in the BC patients, with correlation coefficient *r* = −0.43 (*p* < 0.000; as in **Figure 3B**). This negative correlation is shown, as well, in different BC subgroups, as illustrated in **Table 6**. These data suggest that LINC00511 serves as a molecular sponge to the miR-185-3p. Therefore, the LINC00511/miR-185-3p signaling axis is useful for future BC drug designs.

**Table 6:** Spearman correlation coefficient among the combined investigated ncRNAs expression in BC group and various BC subgroups.

Subgrouping Characteristics	*n*		%	Correlation Coefficient	LINC00511 and miR-185-3p	LINC00511 and miR-301a-3p	miR-185-3p and miR-301a-3p
**Breast Cancer**	70		100	Spearman	−0.438**	0.034	−0.01
				*p-*value	0.000	0.779	0.93
**Age >50 years**	31		44.3	Spearman	−0.369^*^	0.025	−0.231
				*p-*value	0.041	0.895	0.210
**IDC subtype**	59		84.2	Spearman	−0.412^**^	0.115	−0.017
				*p-*value	0.001	0.388	0.900
**+ve ER**	38		54.3	Spearman	−0.483^*^	0.056	−0.046
				*p-*value	0.002	0.739	0.784
**-ve ER**	32		45.7	Spearman	−0.480^*^	0.127	0.040
				*p-*value	0.005	0.487	0.828
**+ve PR**	50		71.4	Spearman	−0.344^*^	0.169	−0.035
				*p-*value	0.014	0.242	0.811
**-ve PR**	20		28.6	Spearman	−0.706**	−0.123	0.015
				*p-*value	0.001	0.607	0.95
**High Ki-67**	10		14.2	Spearman	−0.385	0.209	−0.291
				*p-*value	0.194	0.494	0.334
**+ve HER-2/neu**	23		32.9	Spearman	−0.599^**^	0.079	−0.069
				*p-*value	0.0001	0.653	0.692
**Localized stage III**	34		48.6	Spearman	−0.648^**^	−0.087	−0.005
				*p-*value	0.0001	0.627	0.980
**+ve Pt. Surgical History**	30		42.9	Spearman	−0.352^*^	−0.237	0.028
				*p-*value	0.05	0.208	0.882
**Luminal like subtype**	51		72.8	Spearman	−0.341^*^	0.153	0.022
				*p-*value	0.014	0.282	0.879
**D.M**	9		12.9	Spearman	−0.667^*^	−0.367	−0.033
				*p-*value	0.050	0.332	0.932
**TLC > 5 × 10^3^ cell/µl**	45		64.3	Spearman	−0.420^*^	0.124	−0.220
				*p-*value	0.004	0.416	0.147
**Hb > 10 g/dl**	16		22.9	Spearman	−0.421^*^	−0.034	−0.027
				*p-*value	0.002	0.810	0.844
**Platelet > 150 × 10^3^ cell/µl**	58		82.8	Spearman	−0.437^*^	0.080	−0.126
				*p-*value	0.001**	0.553	0.345
**CEA >1.66 ng/ml**	53	75.7		Spearman	−0.50	0.022	−0.167
				*p-*value	0.001**	0.887	0.272
**CA15-3 > 17.2 U/ml**	49	70		Spearman	−0.524	0.011	0.076
				*p-*value	0.0001**	0.93	0.596

All data were expressed as (n, %) and Spearman correlation coefficient (r) was calculated using SPSS software, *Significant statistical difference less than 0.05, **Significant statistical difference less than or equal 0.001. [D.M, Diabetes mellitus; ER, Estrogen receptor; Hb, Hemoglobin count; HER-2, Human epidermal growth factor receptor-2; IDC, Invasive ductal carcinoma; Ki-67, Proliferative index; PR, Progesterone receptor; TLC, Total leucocyte count].

### Correlation Coefficient Between BC Classical TMs (CEA and CA15-3) and the Studied ncRNAs

The Spearman correlation coefficient among the BC patient’s cohort population is seen in **Supplementary Table S3**. There was a strong positive association between CEA and LINC00511 (*r* = 0.503, *p* = 0.040), but a substantial negative correlation between CA15-3 and miR-185-3p (*r*= −0.705, *p* = 0.023), with no other meaningful associations.

### Correlation Between Protein-Based TMs and ncRNA Expressions in Respect to the BC Patients’ Clinicopathological Factors

The correlation between LINC00511 and miR-185-3p in respect to age > 50 years, IDC subtype, both positive ER status and negative ER status, positive or negative PR status, as well as positive HER-2/neu receptor was significantly negative. LINC00511 showed a negative correlation with miR-185-3p in BC late stages and BC patients with DM. CBC parameters were also assessed and showed significant correlation-related TLC, Hb, and platelet count. Detailed information is provided in **Table 6**. However, LNM, early stage, low grades I/II, other BC subtypes, family history, hormonal contraceptive intake, and CVD all were non-significant.

## Discussion

It is worth noting that BC screening with mammography has been carried out for 3 years nationwide, as part of the Egyptian National Presidential Program/Campaign to promote good health and better life quality, ensuring SDG#3 of good health. This was done in an attempt to deal with one of the most devastating cancers, BC, being the second leading cause of cancer mortality in women with solid tumors (47). Invasive BC accounts for about 80% of BC (48) cases, exhibiting a high heterogeneous nature on both the clinicopathological and the molecular levels, driven by non-genetic, genetic, and/or epigenetic alterations (7). These later alterations emphasize the importance of an early better diagnostic tool/biomarker to increase patients’ survival, together with more effective therapeutic option(s).

In addition to, the difficulty encountered during collecting BC tissue samples, relying on blood biomarker(s) for BC diagnosis remains the gold standard, aiming for an early/better diagnosis (49). Unfortunately, to date, no specific serum biomarker is known particularly for “early BC detection”. Therefore, classical protein markers and novel molecular biomarkers, alone or combined together, may reveal a better picture of early-stage cancer (50). Moreover, mammography and MRI, in combination with serological protein TMs, do not seem to be adequate for cancer detection. Therefore, our study was set to delineate the role of serum protein TMs (CEA and CA15-3) in addition to LINC00511, miR-185-3p, and miR-301a-3p as molecular biomarkers for better clinical BC diagnosis and to correlate their expression levels and the patients’ clinicopathological features. This is the first study, to the best of our knowledge, that focuses on the serum expression levels of LINC00511, miR-185-3p, and miR-301a-3p in peripheral blood, pooled from BC Egyptian female patients, rather than from a cell line and/or tissue samples, to assess the clinical outcome and, as stated earlier, to evaluate their role as better diagnostic markers in BC patients, compared to classical TMs.

In this study, CEA and CA15-3 serum levels were higher in BC patients than in healthy controls at *p* = 0.155 and *p* < 0.001, respectively. The corresponding AUC for CEA and CA15-3 was 0.759 and 0.811, with *p-*values = 0.01 and <0.001, respectively. The cutoff values were 1.66 ng/ml and 17.2 U/ml for CEA and CA15-3, respectively, with a sensitivity of 76.47%, 60.00 and a specificity of 90.0%, 100.0, respectively (**Table 3**). The current cohort indicated that CEA and CA15-3 TMs combination did not achieve better diagnostic efficiency in BC patients, with AUC difference = 0.16 and a *p*-value of 0.13, which was not different from either TM alone in other previous studies (51–52). 

### Clinical Data

The mean onset age of our population was around 50 years, with 55.7% of BC patients being below 50 years, which is slightly younger than the previously recorded/published age (53). This observation might be due to the environmental factors. In our study, only 22.9% had a family history of BC. The average tumor size was around 2 cm, and the LNM was seen in 48.6% of the BC cases. Numerous studies have shown that the delay in BC detection is related to the advanced clinical stage (54, 55). In the current research, the most prevalent BC pathological type was IDC, which is 84.2% of all BC patients. Furthermore, the majority of the participating BC population were grade I/II (78.6%), which was intentionally selected, to serve the research main aim of focusing on early diagnosed BC patients. Data from another study on Egyptian BC patients (12) revealed a reasonably stable hormonal distribution; on the contrary, our study showed 54.3% ER, 71.4% PR positive, and 67.1% exhibiting negative HER-2/neu expression, as well as a higher prevalence of TNBC patients than in the European American population (56). These results could be helpful in deciding a personalized treatment plan for an Egyptian BC patient cohort, different from the European American population. As previously recommended in clinical trials, BC patients with positive ER status are usually treated with an ER inhibitor, such as tamoxifen (TAM), with positive outcome, while patients with negative ER status have a poor prognosis and a greater risk of being hormone-insensitive and immune to TAM (52). Our findings revealed that Egyptian BC patients may have more adverse molecular tumor characteristics, where the luminal-like BC subtype (72.8%) is the most current frequent subtype, followed by HER-2/neu overexpression (22.8%), and finally, TNBC (4.4%) is the least common. This is in agreement with a previous Egyptian study (57) reporting similar percentages.

LncRNAs are a novel type of ncRNAs that are larger than 200 nucleotides long and lack a well specified open reading frame (15). They are important regulatory factors during cancer development. LncRNA dysregulation is linked to the progression of multiple cancers (15), including BC. The exact role of LINC00511 in BC tumorigenesis *in vivo*/clinically is still undisclosed. The current study focused on the clinical evaluation of serum LINC00511 expression levels, being linked to the BC patients’ clinicopathological features, where LINC00511 serum expression demonstrated a 312-fold overexpression in BC patients relative to the healthy controls. LINC00511 expression was positively linked to early BC stages (I/II), with a 2.5-fold rise relative to the primary localized advanced stage III cases, in approximately the same number of BC patients, emphasizing the importance of measuring LINC00511 for BC early diagnosis. It is noteworthy that LINC00511 expression levels are strongly associated with BC disease aggressiveness, being expressed more in the advanced histologic grade (III/IV), positive ER/PR, positive LNM, and tumor size > 2 cm by 2.15-, 1.70-, 1.85-, 6.31-, and 5.32-fold, respectively (**Table 5**). This highlights LINC00511’s significance in assessing disease aggressiveness. These *in vivo* clinical results are consistent with a previous *in vitro* study (30), suggesting that LINC00511 uses as a serum molecular biomarker for BC diagnosis, and contributing to downstream genes transcriptional regulation as well as an increased BC cell growth and expression.

ROC curve analysis revealed that LINC00511 AUC was significantly greater than any other marker, under evaluation in the current study, at 0.980 with a *p*-value < 0.0001. The cutoff value was 0.97-fold change, with a sensitivity of 91.43% and an absolute specificity (100%) (**Table 3**). As a consequence, our results encourage integrating LINC00511+CEA or LINC00511+CA15-3 use as diagnostic markers, *via* increasing BC diagnostic efficiency. The AUC difference was 0.23 and 0.17, respectively, which was better than either CEA or CA15-3 alone (**Figure 1**), with an improved significance to 0.0006 and 0.0000, respectively. To the best of our knowledge, this will be the first research to report the diagnostic efficacy of LINC00511 with CA15-3, in combination, for a better and, hopefully, early diagnosis of clinical BC. A previous study reported LINC00511 upregulation as a BC growth and metastasis contributing factor (30). Our results confirmed LINC00511 to have a major function in clinical BC incidence and proliferation.

MicroRNAs, short (20–24 nt) ncRNAs, play a critical role in post-transcriptional gene expression control in eukaryotic cells *via* affecting mRNA stability and translation and, therefore, play a role in various cancer types (58). miRNA expression patterns change during cancer initiation and development (59). Circulating miRNAs have emerged as a promising novel type of innovative cancer molecular biomarkers, due to their ability to regulate gene expression levels, after targeting mRNA degradation and/or suppressing mRNA translation (60). In the current study, miR-185-3p and miR-301a-3p, the tumor suppressor and the oncogenic miRNAs, respectively, were explored if they were involved in BC incidence or not, or if they could be used as small signature diagnostic biomarkers or not.

The majority of miR-185 research has focused on miR-185-5p, a tumor suppressor miR that has been linked to various forms of cancer progression such as proliferation, apoptosis escape or cell cycle, and chemoresistance (61). Moreover, miR-185-5p was found to inhibit colorectal cancer cell metastasis and invasion (62). Few studies have been performed on the role of miR-185-3p as a tumor suppressor miR first, and in BC patients, second.

**Figure 4:**
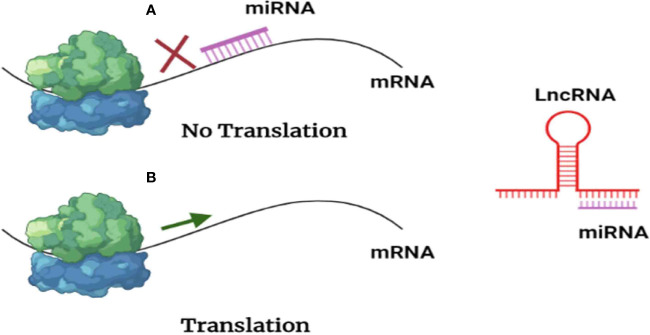
Scheme presenting lncRNAs reversing the negative regulation exerted by miRNAs. **(A)** miRNAs inhibit translation by interacting with messenger RNA. **(B)** As lncRNAs act as a sponge for miRNAs, messenger RNA (mRNA) translation is permitted.

According to the “ceRNA hypothesis”, lncRNAs control target gene expression by competitively binding with miRNAs at the post-transcriptional level (**Figure 4**), creating a massive ceRNA regulatory network. Most studies revealed a dynamic balance between ceRNAs and miRNAs; however, irregular expression of lncRNA disrupts the ceRNA network’s equilibrium, which has been linked to tumorigenesis (63), as sketched in **Figure 4**, where BC would be, hopefully, treated with anti-lncRNA drugs, one of the ncRNA-based cancer therapies (epi-drugs).

Note that BC is a series of biological cascades, originating from intracellular and intercellular components, and is a multifactorial disease (4). In these circumstances, an early diagnostic signature, addressing multiple molecular biomarkers, may have greater strength (50), with a promise for precise medicine implementation, *via* adjusting clinical diagnosis/management, according to specific molecular profiling (50). Based on the ceRNA “sponge” mechanism principle, LINC00511 was previously observed to be upregulated in cell lines (30), and our findings in BC patients’ sera supported these findings. For BC patients, there is a reciprocal expression pattern between LINC00511 and its downstream miR-185-3p (*p* = 0.0002; **Figure 1A**), in which LINC00511 high serum expression levels are associated with low miR-185-3p expression level, as shown in **Figures 2B, F, H**. Using Spearman correlation, a strong negative correlation was found between LINC00511 and miR-185-3p in BC patients (*n* = 70), with *r* = −0.43 (*p* = 0.000; **Figure 3B**). We speculated a 74% sequence complementary between the two sequences at position 525–543 of the annotated LINC00511 (**Figure 3A**), thus validating “The Sponge Theory”.

ROC curve analysis revealed that miR-185-3p had a sensitivity of 95.65% at an accuracy of 87.5%, outperforming all other examined biomarkers. As a result, our findings suggest that combining miR-185-3p and CEA improves the early diagnostic performance in BC patients, with an AUC 0.92, which was slightly higher than CEA or miR-185-3p alone (**Figure 1B**).

As most studies stated, miR-301a was established as an oncogenic miR (onco-miR), which is typically upregulated in cancer, suppressing the expression of potential tumor suppressor genes and ultimately contributing to cancer (40). miR-301a also exerts a significant role in both *in vitro* and *in vivo* tumor cell invasion and migration (64), cell growth or cell death inhibition (38), and chemosensitivity enhancement in BC (65).

According to the study by Ma et al. (66), miR-301a exerted its oncogenic role in BC by deactivating the phosphatase tumor suppressor gene phosphatase and tensin homolog (PTEN), further stimulating the canonical pathway of gene transcription Wingless/Integrated/cadherin-associated protein beta 1 (Wnt/β-catenin), thus establishing a connection between miR-301a expression and BC poor clinical outcome.

In the current research, we measured miR-301a-3p expression levels in serum samples from the first diagnosed 70 female Egyptian BC patients, as well as evaluated their clinicopathological and diagnostic value/utility. As proven in a prior study (64), miR-301a-3p expression elevated levels were substantially associated with BC patients relative to controls and have a positive response to larger BC tumor sizes, suggesting a closely linked correlation between miR-301a-3p expression levels and BC disease virulence.

Zheng et al. (64) revealed ER as a miR-301a-3p direct target. When miRNA expression profiles from ER-positive or ER-negative primary BC were compared, miR-301a-3p was reported to be around 1.5-fold more expressed in ER-negative patients than in ER-positive patients. ER-positive BC cells showed ectopic miR-301a-3p to reduce ER mRNA and ER protein levels, modulating ER target gene expression (64), resulting in ER independence. This could explain the high rate of drug resistance in Egyptian BC patients. Similarly, the effect of hormonal contraceptive use, particularly estrogen pills/hormonal intrauterine implants, had dramatically reduced the expression level of miR-301a-3p, suggesting that both estrogen contraceptives and miR-301a-3p share ER as a potential target.

### Shortcomings (Limitations)

The study does not evaluate the reliability of ncRNAs’ diagnostic performance in BI-RADS-IV BC suspected patients, together with the moderate patient cohort.

### Ongoing Studies

Our research group is currently investigating LINC00511 SNPs in a larger cohort of female Egyptian BC patients. Second, we measure the miR-185-3p transcriptional target, E2F1 mRNA, in a prospective multidisciplinary study, in relation to cancer subtypes, stage, and grade, in order to link obtained results with the clinicopathological features and the clinical outcome. Finally, exosomal ncRNAs are being studied in BC as well.

### Summary

LINC00511, which was highly expressed in BC patients’ sera, is an oncogenic ncRNA. By complementarity binding with 74% matching, LINC00511 would serve as a “sponge” for the tumor suppressor miR-185-3p, to control its expression by BC cells. We believe that our current research highlighted the LINC00511/miR-185-3p axis during early BC incidence. In other words, the current research considered LINC00511 to function as a ceRNA to influence BC incidence.

### Conclusion

Our study found that LINC00511 and miR-301a-3p levels were elevated in Egyptian female BC patients’ liquid biopsy (sera). Furthermore, integrating the detection of LINC00511/CA15-3, LINC00511/CEA, and miR-185-3p/CEA has a superior diagnostic efficiency in recognizing BC patients from healthy individuals than either parameter alone. Elevated serum LINC00511 expression is strongly linked to better early BC diagnosis. Serum LINC00511 and miR-301a-3p may be used as promising molecular biomarkers for better BC diagnosis, as their expression was linked to tumor size, grade, and hormonal receptor status. While LINC00511/miR-185-3p can aid in BC diagnosis/prognosis, further research is needed to calculate serial shifts in serum ncRNA levels at various time points to validate their therapeutic utility in BC.

### Future Prospective

Variation in lncRNA serum expression levels or lncRNA regulatory gene(s)/protein(s) in relation to metastasis/advanced localized BC needs to be examined in relation to cancer immune system to complete the picture. Furthermore, a possible opportunity of investigating the role of the current LINC00511/miR-185-3p axis before and after a (neo)adjuvant therapy or mastectomy needs to be considered.

## Data Availability Statement

The original contributions presented in the study are included in the article/**Supplementary Material**. Further inquiries can be directed to the corresponding author.

## Ethics Statement

From October 2019 to November 2020, this case-controlled retrospective study was held at the Faculty of Pharmacy, Ain Shams University, Biochemistry Department Advanced Biochemistry Research Lab (ABRL), Egypt. Ethical approvals were obtained from both the National Cancer Institute’s (NCI), Cairo University, ethical committee and Ain Shams University, Faculty of Pharmacy’s review board Research Ethical Committee approval (REC ID 259, Date: September 26, 2019). The study was carried out according to the Declaration of Helsinki Guidelines (World Medical Association WMA Declaration of Helsinki: ethical principles for medical research involving human subjects, October 2013, revised July 2018), where all participating individuals (controls or diseased) had signed a written informed consent. The patients/participants provided their written informed consent to participate in this study.

## Author Contributions

ES and MM designed the study. MM and RE collected the clinical samples. ES and MM performed statistical analysis, prepared the tables and figures, drafted the manuscript, and critically read the manuscript. NH designed and supervised the study, critically created tables and figures, curated statistical analysis, drafted, and critically revised the manuscript till submission and publication. All authors contributed to the article and approved the submitted version.

## Funding

This work was minimally supported by the Biochemistry Department Faculty of Pharmacy.

## Conflict of Interest

The authors declare that the research was conducted in the absence of any commercial or financial relationships that could be construed as a potential conflict of interest.

## Publisher’s Note

All claims expressed in this article are solely those of the authors and do not necessarily represent those of their affiliated organizations, or those of the publisher, the editors and the reviewers. Any product that may be evaluated in this article, or claim that may be made by its manufacturer, is not guaranteed or endorsed by the publisher.

## Supplementary Material

Supplementary Table S1 LINC00511, miR-185-3p and miR-301a-3p expression and the classical tumor markers (TMs) correlation with demographic and clinicopathological features within BC patients.

Supplementary Table S2 Multiple regression analysis for predicting the factors affecting LINC00511, miR-185-3p or miR-301a-3p expression levels as dependent variables.

Supplementary Table S3 Correlation coefficient between classical tumor markers (TMs) and the investigated ncRNAs among the BC group.

Supplementary Figure S1 The correlation between LINC00511, miR-185-3p and miR-301a-3p expression in BC subgroups. Spearman correlation coefficient (*r*) was calculated using SPSS software, * Statistical significance less than 0.05. [Hb, Hemoglobin count; HER-2, Human epidermal growth factor receptor-2; TLC, Total leucocyte count].

## Abbreviations

AUC, area under the curve; BC, breast cancer; BMI, body mass index; Bp, base pair; CA15-3, cancer antigen 15-3; CBC, complete blood count; cDNA, complementary DNA; CEA, carcinoembryonic antigen; ceRNA, competitive endogenous RNA; ER, estrogen receptor; Hb, hemoglobin; HER2/neu, human epidermal growth receptor; IDC, invasive ductal carcinoma; LINC00511, long intergenic non-coding RNA 00511; LncRNAs, long non-coding RNA; LNM, lymph node metastasis; LR, likelihood ratio; miRNAs, microRNAs; NCI, National Cancer Institute; ncRNA, non-coding RNA; NPV, negative predictive value; Nt, nucleotides; PPV, positive predictive value; PR, progesterone receptor; qPCR, real-time polymerase chain reaction; ROC, receiver operating characteristic; TAM, tamoxifen; TLC, total leucocyte count; TNBC, triple-negative breast cancer.

## References

[1] Siegel R Ma J Zou Z Jemal A. Cancer Statistics, 2014. CA Cancer J Clin (2014) 64:9–29. doi: 10.3322/caac.21208 24399786

[2] Ma J Jemal A. Breast Cancer Statistics. Breast Cancer Metastasis Drug Resist (2013), 1–18. doi: 10.1007/978-1-4614-5647-6_1

[3] Omar S Khaled H Gaafar R Zekry AR Eissa S El Khatib O. Breast Cancer in Egypt: A Review of Disease Presentation and Detection Strategies. EMHJ-Eastern Mediterr Heal J (2003) 9(3):448–63. 15751939

[4] Eiro N Gonzalez LO Fraile M Cid S Schneider J Vizoso FJ. Breast Cancer Tumor Stroma: Cellular Components, Phenotypic Heterogeneity, Intercellular Communication, Prognostic Implications and Therapeutic Opportunities. Cancers (Basel) (2019) 11:664. doi: 10.3390/cancers11050664 PMC6562436 31086100

[5] Kanwal R Gupta S. Epigenetic Modifications in Cancer. Clin Genet (2012) 81:303–11. doi: 10.1111/j.1399-0004.2011.01809.x PMC3590802 22082348

[6] Oh HJ Chung J-K Kang JH Kang WJ Noh DY Park IA. The Relationship Between Expression of the Sodium/Iodide Symporter Gene and the Status of Hormonal Receptors in Human Breast Cancer Tissue. Cancer Res Treat Off J Korean Cancer Assoc (2005) 37:247. doi: 10.4143/crt.2005.37.4.247 PMC2785920 19956522

[7] Baliu-Piqué M Pandiella A Ocana A. Breast Cancer Heterogeneity and Response to Novel Therapeutics. Cancers (Basel) (2020) 12:3271. doi: 10.3390/cancers12113271 PMC7694303 33167363

[8] Cajal SR Sesé M Capdevila C Aasen T De Mattos-Arruda L Diaz-Cano SJ. Clinical Implications of Intratumor Heterogeneity: Challenges and Opportunities. J Mol Med (2020) 98:161–77. doi: 10.1007/s00109-020-01874-2 PMC7007907 31970428

[9] Sun B Liu C Li H Zhang L Luo G Liang S. Research Progress on the Interactions Between Long non−Coding RNAs and microRNAs in Human Cancer. Oncol Lett (2020) 19:595–605. doi: 10.3892/ol.2019.11182 31897175 PMC6923957

[10] Zhou S He Y Yang S Hu J Zhang Q Chen W. The Regulatory Roles of lncRNAs in the Process of Breast Cancer Invasion and Metastasis. Biosci Rep (2018) 38(5). doi: 10.1042/BSR20180772 PMC6165837 30217944

[11] Jiang N Zhang X Gu X Li X Shang L. Progress in Understanding the Role of lncRNA in Programmed Cell Death. Cell Death Discovery (2021) 7:1–11. doi: 10.1038/s41420-021-00407-1 PMC7870930 33558499

[12] Youness RA Hafez HM Khallaf E Assal RA Abdel Motaal A Gad MZ. The Long Noncoding RNA sONE Represses Triple-Negative Breast Cancer Aggressiveness Through Inducing the Expression of miR-34a, miR-15a, miR-16, and Let-7a. J Cell Physiol (2019) 234:20286–97. doi: 10.1002/jcp.28629 30968427

[13] Lopez-Pajares V. Long non-Coding RNA Regulation of Gene Expression During Differentiation. Pflügers Arch J Physiol (2016) 468:971–81. doi: 10.1007/s00424-016-1809-6 26996975

[14] Böhmdorfer G Wierzbicki AT. Control of Chromatin Structure by Long Noncoding RNA. Trends Cell Biol (2015) 25:623–32. doi: 10.1016/j.tcb.2015.07.002 PMC4584417 26410408

[15] Guo X Hua Y. CCAT1: An Oncogenic Long Noncoding RNA in Human Cancers. J Cancer Res Clin Oncol (2017) 143:555–62. doi: 10.1007/s00432-016-2268-3 PMC11819113 27638771

[16] Fan L Huang C Li J Gao T Lin Z Yao T. Long Non−Coding RNA Urothelial Cancer Associated 1 Regulates Radioresistance *via* the Hexokinase 2/Glycolytic Pathway in Cervical Cancer. Int J Mol Med (2018) 42:2247–59. doi: 10.3892/ijmm.2018.3778 30015920

[17] Wang L Duan W Yan S Xie Y Wang C. Circulating Long Non-Coding RNA Colon Cancer-Associated Transcript 2 Protected by Exosome as a Potential Biomarker for Colorectal Cancer. BioMed Pharmacother (2019) 113:108758. doi: 10.1016/j.biopha.2019.108758 30877883

[18] Qiao L Liu X Tang Y Zhao Z Zhang J Liu H. Knockdown of Long Non-Coding RNA Prostate Cancer-Associated ncRNA Transcript 1 Inhibits Multidrug Resistance and C-Myc-Dependent Aggressiveness in Colorectal Cancer Caco-2 and HT-29 Cells. Mol Cell Biochem (2018) 441:99–108. doi: 10.1007/s11010-017-3177-8 28884413

[19] Meng Y Liu Y-L Li K Fu T. Prognostic Value of Long Non-Coding RNA Breast Cancer Anti-Estrogen Resistance 4 in Human Cancers: A Meta-Analysis. Med (Baltimore) (2019) 98(21):e15793. doi: 10.1097/MD.0000000000015793 PMC6571273 31124974

[20] Thomson DW Dinger ME. Endogenous microRNA Sponges: Evidence and Controversy. Nat Rev Genet (2016) 17:272–83. doi: 10.1038/nrg.2016.20 27040487

[21] Zhang J Sui S Wu H Zhang J Zhang X Xu S. The Transcriptional Landscape of lncRNAs Reveals the Oncogenic Function of LINC00511 in ER-Negative Breast Cancer. Cell Death Dis (2019) 10:599. doi: 10.1038/s41419-019-1835-3 31395854 PMC6687715

[22] Wang B Wang YZ Wang K Ji TL Cui BZ He YY. Expression and Clinical Significance of lncRNA LINC00511 in Plasma Exosome of Glioma Patients. Chin J Pr Nerv Dis (2019) 22:1548–53.

[23] Wang J Wang SS Ma H. Expressions Clinical Signification and Prognostic Analysis of Linc00511 in Ovarian Cancer Tissues. J Pr Med (2019) 35:2584–91.

[24] Mao B-D Xu P Zhong Y Ding W-W Meng Q-Z. LINC00511 Knockdown Prevents Cervical Cancer Cell Proliferation and Reduces Resistance to Paclitaxel. J Biosci (2019) 44:1–13. doi: 10.1007/s12038-019-9851-0 31180057

[25] Qiao S Qi K Liu C Xu C Ma J Xu X. Long Intergenic non-Coding RNA 511 Correlates With Improved Prognosis, and Hinders Osteosarcoma Progression Both *In Vitro* and *In Vivo* . J Clin Lab Anal (2020) 34:e23164. doi: 10.1002/jcla.23164 31893577 PMC7246352

[26] Sun C-C Li S-J Li G Hua R-X Zhou X-H Li D-J. Long Intergenic Noncoding RNA 00511 Acts as an Oncogene in Non–Small-Cell Lung Cancer by Binding to EZH2 and Suppressing P57. Mol Ther Acids (2016) 5:e385. doi: 10.1038/mtna.2016.94 PMC5155326 27845772

[27] Bridges MC Daulagala AC Kourtidis A. LNCcation: lncRNA Localization and Function. J Cell Biol (2021) 220:e202009045. doi: 10.1083/jcb.202009045 33464299 PMC7816648

[28] Noh JH Kim KM McClusky WG Abdelmohsen K Gorospe M. Cytoplasmic Functions of Long Noncoding RNAs. Wiley Interdiscip Rev RNA (2018) 9:e1471. doi: 10.1002/wrna.1471 29516680 PMC5963534

[29] Sun Q Hao Q Prasanth KV. Nuclear Long Noncoding RNAs: Key Regulators of Gene Expression. Trends Genet (2018) 34:142–57. doi: 10.1016/j.tig.2017.11.005 PMC6002860 29249332

[30] Lu G Li Y Ma Y Lu J Chen Y Jiang Q. Long Noncoding RNA LINC00511 Contributes to Breast Cancer Tumourigenesis and Stemness by Inducing the miR-185-3p/E2F1/Nanog Axis. J Exp Clin Cancer Res (2018) 37:1–11. doi: 10.1186/s13046-018-0945-6 30482236 PMC6260744

[31] Xu S Kong D Chen Q Ping Y Pang D. Oncogenic Long Noncoding RNA Landscape in Breast Cancer. Mol Cancer (2017) 16:1–15. doi: 10.1186/s12943-017-0696-6 28738804 PMC5525255

[32] Huntzinger E Izaurralde E. Gene Silencing by microRNAs: Contributions of Translational Repression and mRNA Decay. Nat Rev Genet (2011) 12:99–110. doi: 10.1038/nrg2936 21245828

[33] Negrini M Calin GA. Breast Cancer Metastasis: A microRNA Story. Breast Cancer Res (2008) 10:1–4. doi: 10.1186/bcr1867 PMC2397516 18373886

[34] Jalali S Bhartiya D Lalwani MK Sivasubbu S Scaria V. Systematic Transcriptome Wide Analysis of lncRNA-miRNA Interactions. PloS One (2013) 8:e53823. doi: 10.1371/journal.pone.0053823 23405074 PMC3566149

[35] Xu J Chen G Zhang Y Huang Z Cheng X Gu H. LINC00511 Promotes Osteosarcoma Tumorigenesis and Invasiveness Through the miR-185-3p/E2F1 Axis. BioMed Res Int (2020) 1974506. doi: 10.1155/2020/1974506 PMC7501572 32964019

[36] Zhao L Zhang Y Liu J Yin W Jin D Wang D. miR-185 Inhibits the Proliferation and Invasion of non-Small Cell Lung Cancer by Targeting KLF7. Oncol Res Featur Preclin Clin Cancer Ther (2019) 27:1015–23. doi: 10.3727/096504018X15247341491655 PMC7848451 29716672

[37] Yurikova OY Aisina DE Niyazova RE Atambayeva SA Labeit S Ivashchenko AT. The Interaction of miRNA-5p and miRNA-3p With the mRNAs of Orthologous Genes. Mol Biol (2019) 53:612–23. doi: 10.1134/S0026893319040174 31397443

[38] Fang Y Sun B Xiang J Chen Z. MiR-301a Promotes Colorectal Cancer Cell Growth and Invasion by Directly Targeting SOCS6. Cell Physiol Biochem (2015) 35:227–36. doi: 10.1159/000369690 25591765

[39] Chen Z Chen L Dai H Wang P Gao S Wang K. miR-301a Promotes Pancreatic Cancer Cell Proliferation by Directly Inhibiting Bim Expression. J Cell Biochem (2012) 113:3229–35. doi: 10.1002/jcb.24200 22628193

[40] Xu X He X Tao H Zhang W Wang Y Ye Z. Abnormal Expression of Mi R-301a in Gastric Cancer Associated With Progression and Poor Prognosis. J Surg Oncol (2013) 108:197–202. doi: 10.1002/jso.23374 23832550

[41] Zhou P Jiang W Wu L Chang R Wu K Wang Z. miR-301a is a Candidate Oncogene That Targets the Homeobox Gene Gax in Human Hepatocellular Carcinoma. Dig Dis Sci (2012) 57:1171–80. doi: 10.1007/s10620-012-2099-2 22373864

[42] Hu Z Dong J Wang L-E Ma H Liu J Zhao Y. Serum microRNA Profiling and Breast Cancer Risk: The Use of miR-484/191 as Endogenous Controls. Carcinogenesis (2012) 33:828–34. doi: 10.1093/carcin/bgs030 22298638

[43] Hammer C Fanning A Crowe J. Overview of Breast Cancer Staging and Surgical Treatment Options. Cleve Clin J Med (2008) 75 Suppl 1:S10–6. doi: 10.3949/ccjm.75.suppl_1.s10 18457192

[44] Bloom HJ Richardson WW. Histological Grading and Prognosis in Breast Cancer; a Study of 1409 Cases of Which 359 Have Been Followed for 15 Years. Br J Cancer (1957) 11:359–77. doi: 10.1038/bjc.1957.43 PMC2073885 13499785

[45] Eliyatkın N Yalçın E Zengel B Aktaş S Vardar E. Molecular Classification of Breast Carcinoma: From Traditional, Old-Fashioned Way to a New Age, and a New Way. J Breast Heal (2015) 11:59. doi: 10.5152/tjbh.2015.1669 PMC5351488 28331693

[46] Livak KJ Schmittgen TD. Analysis of Relative Gene Expression Data Using Real-Time Quantitative PCR and the 2(-Delta Delta C(T)) Method. Methods (2001) 25:402–8. doi: 10.1006/meth.2001.1262 11846609

[47] Bray F Ferlay J Soerjomataram I Siegel RL Torre LA Jemal A. Global Cancer Statistics 2018: GLOBOCAN Estimates of Incidence and Mortality Worldwide for 36 Cancers in 185 Countries. CA Cancer J Clin (2018) 68:394–424. doi: 10.3322/caac.21492 30207593

[48] Sharma GN Dave R Sanadya J Sharma P Sharma KK. Various Types and Management of Breast Cancer: An Overview. J Adv Pharm Technol Res (2010) 1:109. 22247839 PMC3255438

[49] Zubor P Kubatka P Kajo K Dankova Z Polacek H Bielik T. Why the Gold Standard Approach by Mammography Demands Extension by Multiomics? Application of Liquid Biopsy miRNA Profiles to Breast Cancer Disease Management. Int J Mol Sci (2019) 20:2878. doi: 10.3390/ijms20122878 PMC6627787 31200461

[50] Loke SY Lee ASG. The Future of Blood-Based Biomarkers for the Early Detection of Breast Cancer. Eur J Cancer (2018) 92:54–68. doi: 10.1016/j.ejca.2017.12.025 29413690

[51] Shao Y Sun X He Y Liu C Liu H. Elevated Levels of Serum Tumor Markers CEA and CA15-3 are Prognostic Parameters for Different Molecular Subtypes of Breast Cancer. PloS One (2015) 10:e0133830. doi: 10.1371/journal.pone.0133830 26207909 PMC4514648

[52] Li J Liu L Feng Z Wang X Huang Y Dai H. Tumor Markers CA15-3, CA125, CEA and Breast Cancer Survival by Molecular Subtype: A Cohort Study. Breast Cancer (2020) 27:621–30. doi: 10.1007/s12282-020-01058-3 32040723

[53] Bidoli E Virdone S Hamdi-Cherif M Toffolutti F Taborelli M Panato C. Worldwide Age at Onset of Female Breast Cancer: A 25-Year Population-Based Cancer Registry Study. Sci Rep (2019) 9:1–8. doi: 10.1038/s41598-019-50680-5 31575963 PMC6773713

[54] Tartter PI Pace D Frost M Bernstein JL. Delay in Diagnosis of Breast Cancer. Ann Surg (1999) 229:91. doi: 10.1097/00000658-199901000-00012 9923805 PMC1191613

[55] Rossi S Cinini C Di Pietro C Lombardi CP Crucitti A Bellantone R. Diagnostic Delay in Breast Cancer: Correlation With Disease Stage and Prognosis. Tumori J (1990) 76:559–62. doi: 10.1177/030089169007600609 2284692

[56] Jiagge E Jibril AS Chitale D Bensenhaver JM Awuah B Hoenerhoff M. Comparative Analysis of Breast Cancer Phenotypes in African American, White American, and West Versus East African Patients: Correlation Between African Ancestry and Triple-Negative Breast Cancer. Ann Surg Oncol (2016) 23:3843–9. doi: 10.1245/s10434-016-5420-z 27469125

[57] Liu L Zhu Y Liu AM Feng Y Chen Y. Long Noncoding RNA LINC00511 Involves in Breast Cancer Recurrence and Radioresistance by Regulating STXBP4 Expression *via* miR-185. Eur Rev Med Pharmacol Sci (2019) 23:7457–68. doi: 10.26355/eurrev-201909-18855 31539133

[58] He L Hannon GJ. MicroRNAs: Small RNAs With a Big Role in Gene Regulation. Nat Rev Genet (2004) 5:522–31. doi: 10.1038/nrg1379 15211354

[59] Calin GA Croce CM. MicroRNA Signatures in Human Cancers. Nat Rev Cancer (2006) 6:857–66. doi: 10.1038/nrc1997 17060945

[60] O’Bryan S Dong S Mathis JM Alahari SK. The Roles of Oncogenic miRNAs and Their Therapeutic Importance in Breast Cancer. Eur J Cancer (2017) 72:1–11. doi: 10.1016/j.ejca.2016.11.004 27997852

[61] Ma X Shen D Li H Zhang Y Lv X Huang Q. MicroRNA-185 Inhibits Cell Proliferation and Induces Cell Apoptosis by Targeting VEGFA Directly in Von Hippel-Lindau–inactivated Clear Cell Renal Cell Carcinoma. In: Urologic Oncology: Seminars and Original Investigations. Elsevier, China (2015). p. 169–e1. 10.1016/j.urolonc.2015.01.003 25700976

[62] Zhang Z Liu X Feng B Liu N Wu Q Han Y. STIM1, a Direct Target of microRNA-185, Promotes Tumor Metastasis and is Associated With Poor Prognosis in Colorectal Cancer. Oncogene (2015) 34:4808–20. doi: 10.1038/onc.2014.404 PMC4569941 25531324

[63] Li Q Shao Y Zhang X Zheng T Miao M Qin L. Plasma Long Noncoding RNA Protected by Exosomes as a Potential Stable Biomarker for Gastric Cancer. Tumor Biol (2015) 36:2007–12. doi: 10.1007/s13277-014-2807-y 25391424

[64] Zheng J-Z Huang Y-N Yao L Liu Y-R Liu S Hu X. Elevated miR-301a Expression Indicates a Poor Prognosis for Breast Cancer Patients. Sci Rep (2018) 8:2225. doi: 10.1038/s41598-018-20680-y 29396508 PMC5797194

[65] Deng S Zhang T Chen X Shi J Meng M Yue G. Is There a Correlation Between miR-301a Expression and Neoadjuvant Chemotherapy Efficacy in Breast Cancer Tissue? Biochem Biophys Rep (2021) 26:100947. doi: 10.1016/j.bbrep.2021.100947 33614999 PMC7878978

[66] Ma F Zhang J Zhong L Wang L Liu Y Wang Y. Upregulated microRNA-301a in Breast Cancer Promotes Tumor Metastasis by Targeting PTEN and Activating Wnt/β-Catenin Signaling. Gene (2014) 535:191–7. doi: 10.1016/j.gene.2013.11.035 24315818

