# Supplementary material for: Competitive Endogenous Role of the LINC00511/miR-185-3p Axis and miR-301a-3p From Liquid Biopsy as Molecular Markers for Breast Cancer Diagnosis
Source: Front Oncol. 2021 Oct 20;11:749753. doi: 10.3389/fonc.2021.749753 (PMC8567754; doi:10.3389/fonc.2021.749753)
Supplement: Supplementary Table S1 — LINC00511, miR-185-3p and miR-301a-3p expression and the classical tumor markers (TMs) correlation with demographic and clinicopathological features within BC patients. [file DataSheet_1.docx]

**Supplementary Table S1.** LINC00511, miR-185-3p and miR-301a-3p expression and the classical tumor markers (TMs) correlation with demographic and clinicopathological features within BC patients

|  | **Correlation Coefficient** | **ncRNAs** | | | **TMs** | |
| --- | --- | --- | --- | --- | --- | --- |
| **Characteristics** |  | **LINC00511** | **miR-185-3p** | **miR-301a-3p** | **CEA** | **CA15-3** |
| **Age (Years)** | Spearman | 0.117 | 0.111 | 0.026 | -0.105 | -0.058 |
|  | *P* value | 0.336 | 0.361 | 0.832 | 0.387 | 0.632 |
| **BMI (kg/m^2^)** | Spearman | 0.085 | -0.133 | -0.088 | 0.117 | -0.010 |
|  | *P* value | 0.62 | 0.448 | 0.616 | 0.496 | 0.955 |
| **TLC (4x10^3^cell/µL)** | Spearman | -0.147 | 0.031 | -0.048 | 0.127 | 0.111 |
|  | *P* value | 0.307 | 0.833 | 0.743 | 0.380 | 0.444 |
| **Hb (gm/dl)** | Spearman | -0.039 | -0.080 | 0.023 | 0.052 | 0.201 |
|  | *P* value | 0.788 | 0.582 | 0.874 | 0.721 | 0.162 |
| **Platelets (x10^3^cell/µL)** | Spearman | -0.038 | 0.001 | -0.112 | -0.022 | 0.088 |
|  | *P* value | 0.795 | 0.993 | 0.440 | 0.878 | 0.545 |
| **Menopause** | Point-Biserial | 0.043 | 0.163 | 0.106 | -0.099 | -0.143 |
|  | *P* value | 0.725 | 0.178 | 0.380 | 0.417 | 0.238 |
| **Tumor Size (Cm)** | Point-Biserial | 0.067 | 0.138 | 0.095 | -0.074 | 0.096 |
|  | *P* value | 0.613 | 0.298 | 0.473 | 0.578 | 0.468 |
| **LNM** | Point-Biserial | -0.177 | 0.160 | -0.048 | 0.265* | -0.201 |
|  | *P* value | 0.172 | 0.218 | 0.716 | 0.03 | 0.12 |
| **ER-Status** | Point-Biserial | 0.013 | 0.023 | -0.120 | -0.089 | 0.036 |
|  | *P* value | 0.916 | 0.847 | 0.322 | 0.462 | 0.766 |
| **PR-Status** | Point-Biserial | 0.088 | -0.051 | -0.085 | 0.164 | -0.022 |
|  | *P* value | 0.471 | 0.676 | 0.486 | 0.174 | 0.857 |
| **HER-2/neu Status** | Point-Biserial | -0.050 | -0.226 | 0.139 | 0.048 | -0.214 |
|  | *P* value | 0.680 | 0.060 | 0.250 | 0.693 | 0.076 |
| **Ki-67 status** | Point-Biserial | 0.227 | -0.466 | 0.036 | 0.359 | 0.012 |
|  | *P* value | 0.365 | 0.05 | 0.888 | 0.144 | 0.962 |
| **No. of offspring** | Point-Biserial | 0.161 | -0.144 | -0.123 | 0.146 | -0.019 |
|  | *P* value | 0.280 | 0.333 | 0.411 | 0.326 | 0.901 |
| **BC Family History** | Point-Biserial | 0.123 | -0.088 | 0.084 | -0.091 | 0.030 |
|  | *P* value | 0.311 | 0.467 | 0.488 | 0.454 | 0.803 |
| **Pt. Surgical History** | Point-Biserial | -0.042 | 0.128 | -0.304* | -0.024 | 0.016 |
|  | *P* value | 0.730 | 0.292 | 0.011 | 0.844 | 0.898 |
| **Hormonal Contraceptive intake** | Point-Biserial | 0.023 | -0.074 | 0.061 | -0.199 | 0.071 |
|  | *P* value | 0.851 | 0.545 | 0.613 | 0.099 | 0.561 |

**Spearman correlation coefficient *r* was used to measure the degree of association between two continuous non-parametric variables, while point-biserial correlation was used to measure the association that exists between two variables; one continuous and the other is dichotomous using SPSS software.** ***Significant statistical difference less than 0.05**. [BMI, Body mass index; ER, Estrogen receptor; Hb, Hemoglobin count; HER-2, Human epidermal growth factor receptor-2; LNM, Lymph-node metastasis; Ki-67, Proliferative index; PR, Progesterone receptor; TLC, Total leucocyte count; TNBC; Triple negative breast cancer; TNM, Tumor node metastasis.]

**Supplementary Table S2:** Multiple regression analysis for predicting the factors affecting LINC00511, miR-185-3p or miR-301a-3p expression levels as dependent variables

| **Independent Variables** | **Dependent variables** | | | | | |
| --- | --- | --- | --- | --- | --- | --- |
|  | **LINC00511 expression**  F (8,14) = 0.534,  *P*=0.813, *R*2= 0.234 | | **miR-185-3p expression**  F (8,14) = 0.733,  *P=*0.662, *R^2^*= 0.295 | | **miR-301a-3p expression**  F (8,14) = 1.215,  *P=*0.358, *R^2^*= 0.410 | |
|  | Standardized Beta | *P* | Standardized Beta | *P* | Standardized Beta | *P* |
| **Age** (years) | 0.191 | 0.540 | 0.110 | 0.710 | -0.177- | 0.517 |
| **BMI** (kg/m2) | -0.014- | 0.973 | -0.020- | 0.958 | -0.008- | 0.982 |
| **Menopausal Status** | 0.357 | 0.186 | 0.116 | 0.646 | 0.445 | 0.068 |
| **BC Family History** | 0.257 | 0.343 | 0.215 | 0.408 | 0.445 | 0.074 |
| **Pt. Surgical History** | 0.194 | 0.523 | -0.306- | 0.298 | -0.228- | 0.395 |
| **Hemoglobin** (gm/dL) | -0.243- | 0.475 | -0.069- | 0.831 | 0.201 | 0.500 |
| **Platelets** (x103cell/µL) | 0.075 | 0.795 | -0.269- | 0.339 | -0.185- | 0.470 |
| **Number of offspring** | 0.068 | 0.808 | -0.056- | 0.835 | -0.060- | 0.809 |

**Multiple regression analyses were run to investigate the influence of age, BMI, menopausal status, BC family history, st. surgical history, hemoglobin content, platelets count, and** **number of offspring (independent variables) on the expression levels of LINC00511, miR-185-3p or miR-301a-3p as dependent variables using SPSS software.** [BC; breast cancer, BMI; body mass index.]

**Supplementary Table S3.** Correlation coefficient between classical tumor markers (TMs) and the investigated ncRNAs among the BC group

|  | **Correlation coefficient** | **ncRNAs** | | |
| --- | --- | --- | --- | --- |
| **TMs** |  | **LINC00511** | **miR-185-3p** | **miR-301a-3p** |
| **CEA** | *R* | 0.503* | -0.278 | -0.276 |
|  | *P* value | 0.040 | 0.280 | 0.284 |
| **CA15-3** | *R* | 0.600 | -0.705* | -0.200 |
|  | *P* value | 0.067 | 0.023 | 0.580 |

Correlation coefficient was calculated using SPSS software and determined by Spearman correlation *r* test, *Significant statistical difference less than 0.05.


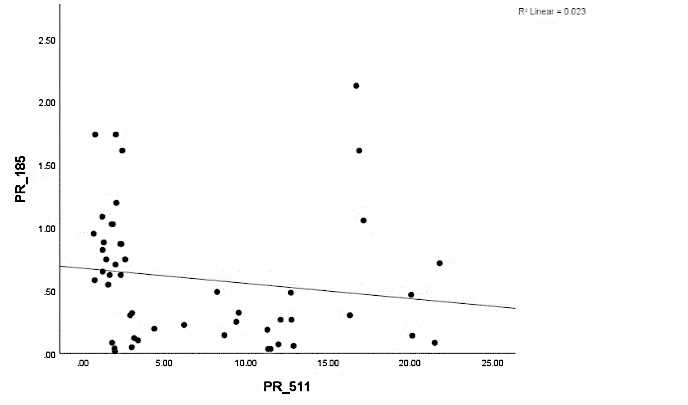


**r = -0.344**

***P* =0.014**

**Positive Progesterone Receptor**

**miR-185-3p**

**LINC00511**


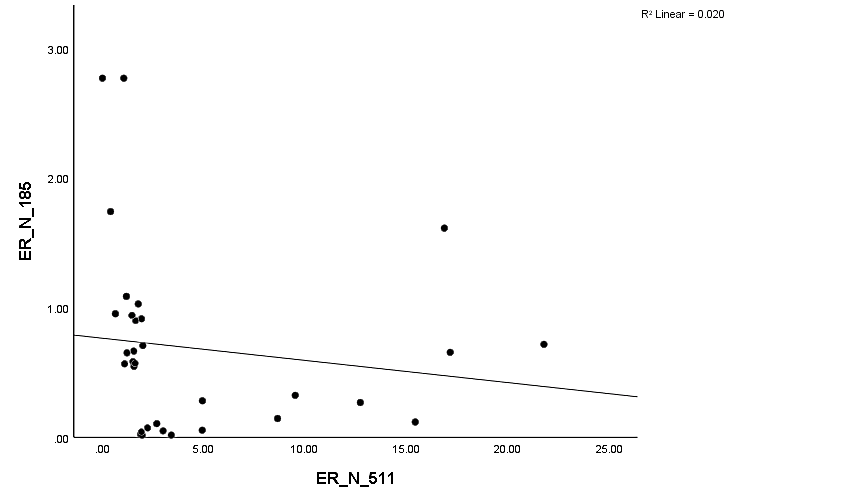


**r = -0.48**

***P* =0.005**

**Negative Estrogen Receptor**

**miR-185-3p**

**LINC00511**


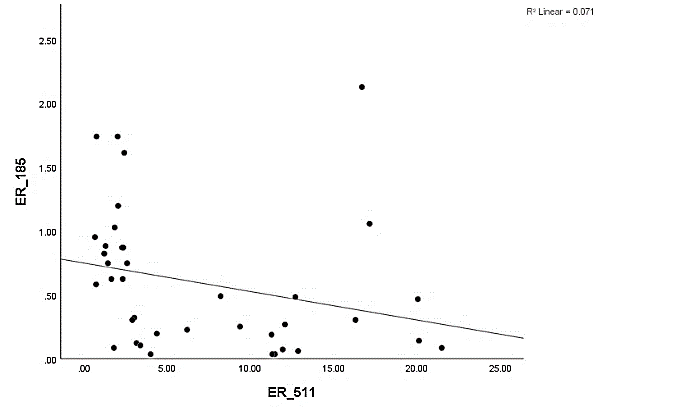


**r = -0.483**

***P* = 0.002**

**Positive Estrogen Receptor**

**miR-185-3p**

**LINC00511**


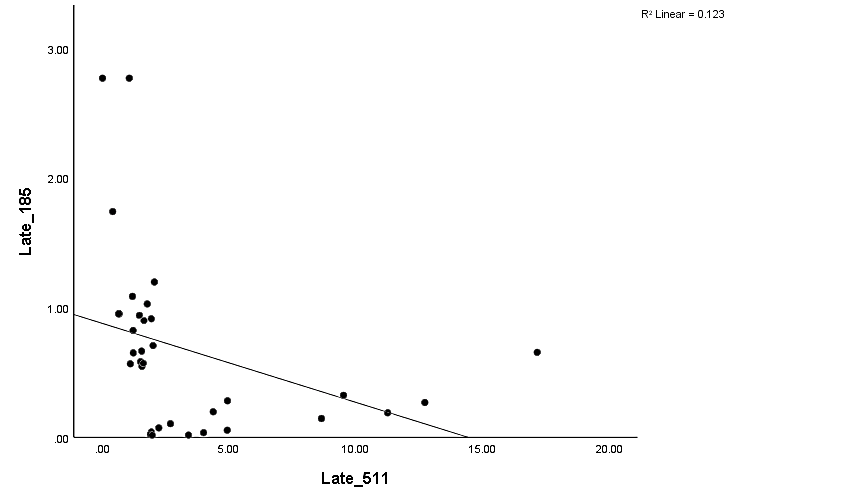


**r = -0.648**

***P* =0.000**

**Late Stage**

**miR-185-3p**

**LINC00511**


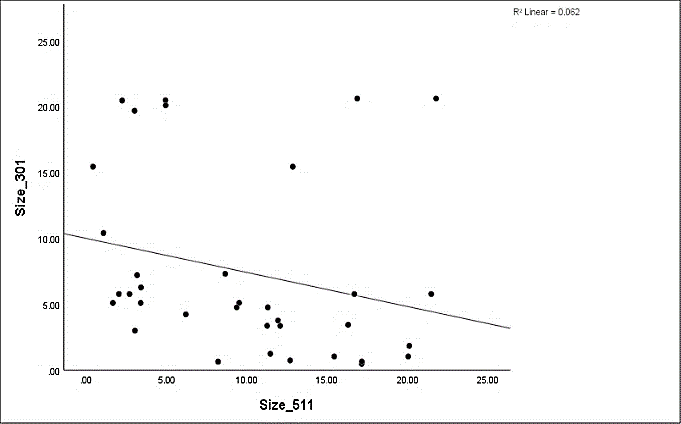


**r = -0.361**

***P* =0.033**

**Tumor Size >2Cm**

**LINC00511**

**miR-301a**


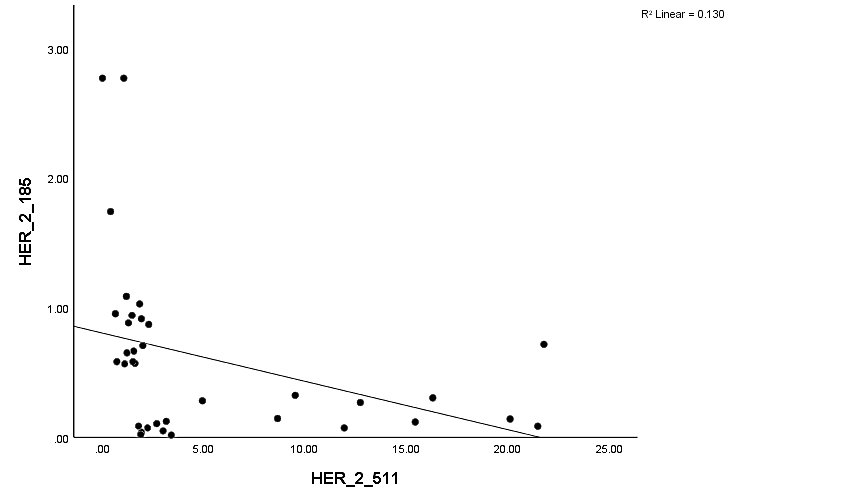


**r = -0.599**

***P* = 0.000**

**Positive HER-2/neu Receptor**

**miR-185-3p**

**LINC00511**


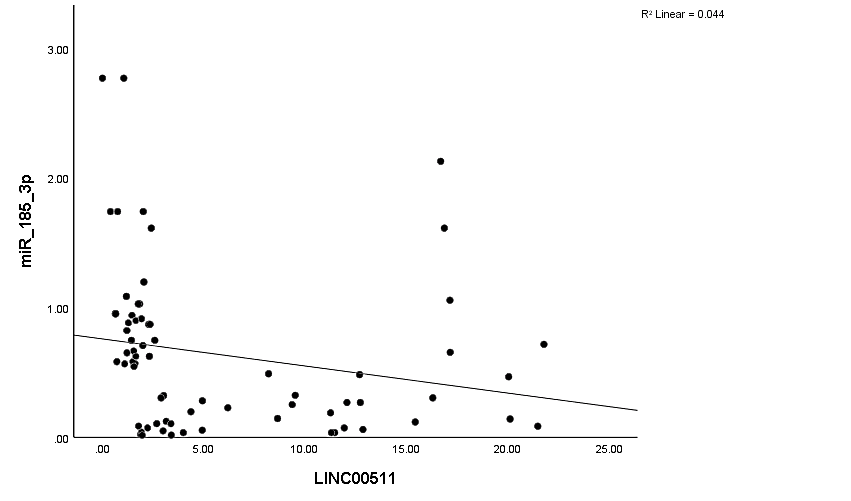


**miR-185-3p**

**r =0.438**

***P* =0.000**

**LINC00511**

**Breast Cancer**


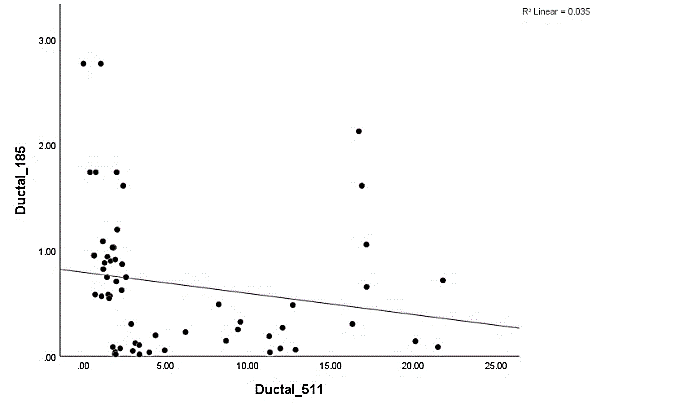


**Ductal Carcinoma Subtype**

**r = -0.412**

***P* =0.001**

**miR-185-3p**

**LINC00511**


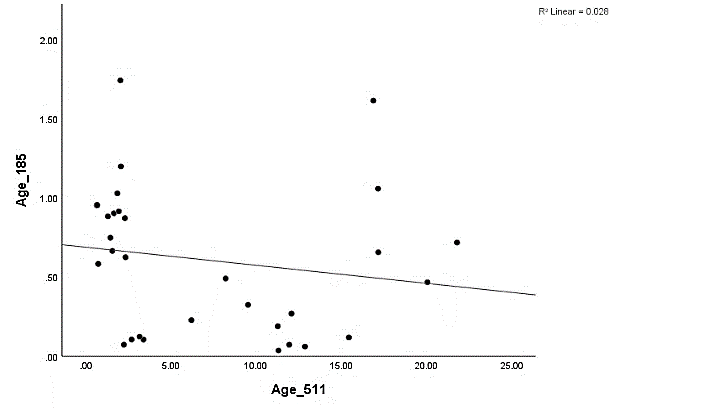


**miR-185-3p**

**Age >50**

**r = -0.369**

***P* = 0.041**

**LINC00511**


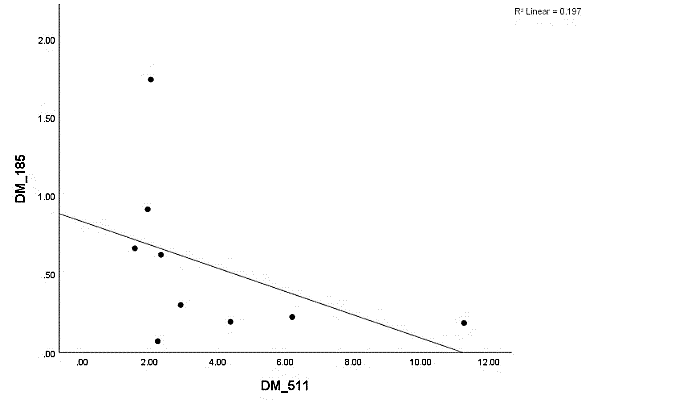


**r = -0.667**

***P* =0.050**

**Diabetes Mellites**

**miR-185-3p**

**LINC00511**


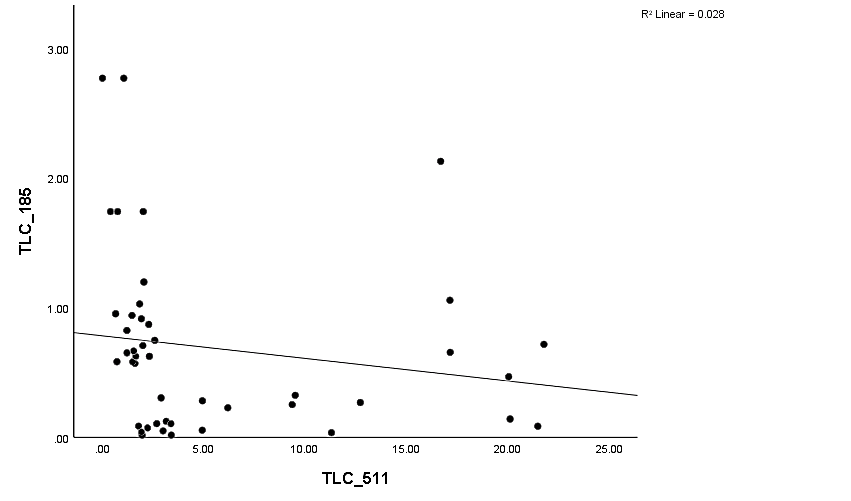


**r = -0.420**

***P* =0.004**

**TLC > 4x10^3^cell/µL**

**miR-185-3p**

**LINC00511**


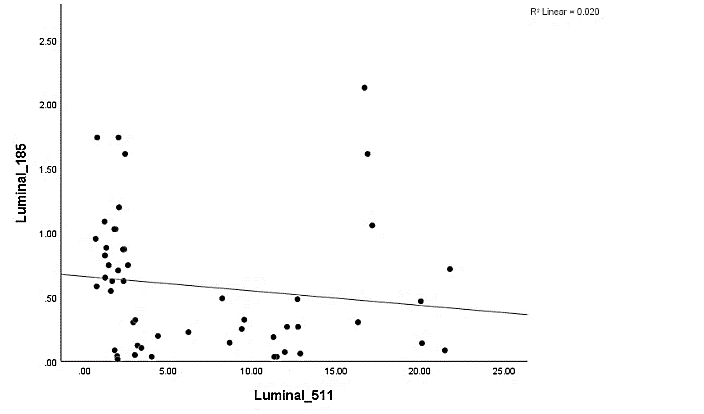


**r = -0.341**

***P* =0.014**

**Luminal Like Subtype**

**miR-185-3p**

**LINC00511**


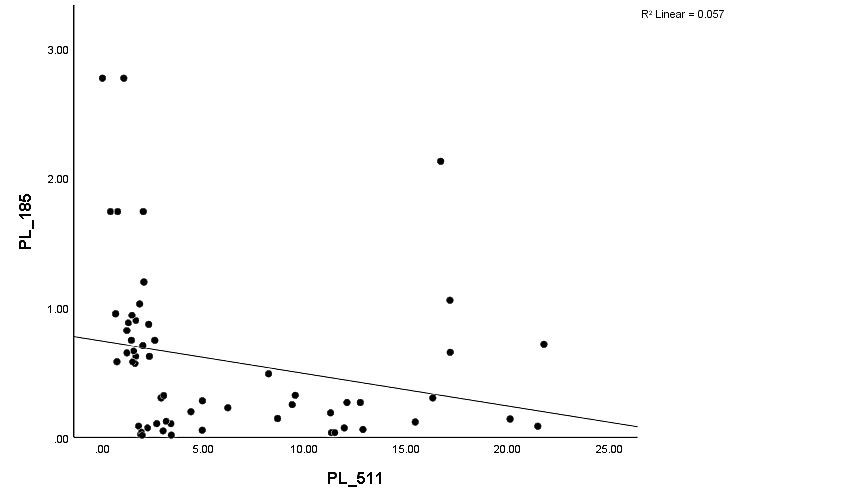


**r = -0.437**

***P* =0.001**

**Platelet > 150 x10^3^cell/µL**

**miR-185-3p**

**LINC00511**


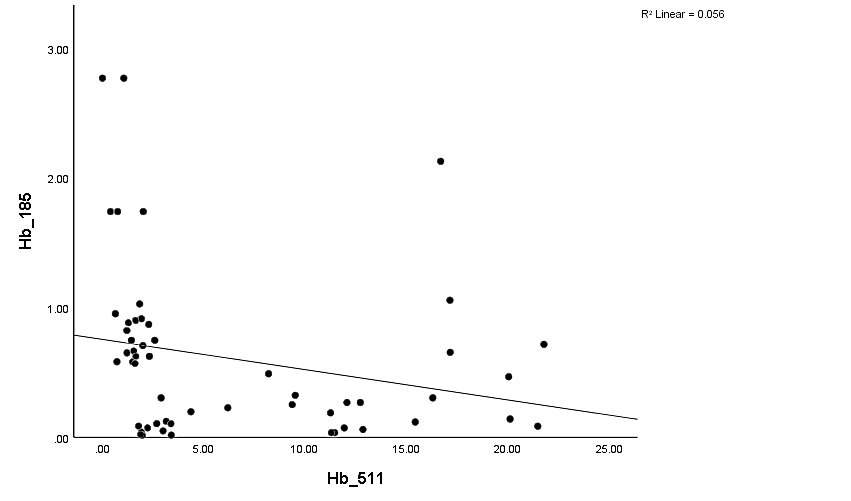


**r = -0.421**

***P* = 0.002**

**Hb > 10gram/dL**

**miR-185-3p**

**LINC00511**

**Supplementary Fig. S1.** The correlation between LINC00511, miR-185-3p and miR-301a-3p expression in BC subgroups. Spearman correlation coefficient (*r)* was calculated using SPSS software, * Statistical significance less than 0.05. **[Hb, Hemoglobin count; HER-2, Human epidermal growth factor receptor-2; TLC, Total leucocyte count.]**
